# Effects of Therapeutic Aquatic Exercise Versus Physical Therapy Modalities on Pain and Disability in People With Chronic Low Back Pain: Potential Mediating Roles of Kinesiophobia, Anxiety, and Depression

**DOI:** 10.1155/prm/5537314

**Published:** 2026-04-12

**Authors:** Shengfeng Liu, Yanan Zheng, Changcheng Chen, Juan Wang, Mengsi Peng

**Affiliations:** ^1^ Department of Rehabilitation Therapy, The Second Affiliated Hospital of Hainan Medical University, Haikou, China, hainmc.edu.cn; ^2^ Department of Rehabilitation Medicine, The First Affiliated Hospital of Anhui Medical University, Hefei, China, ahmu.edu.cn; ^3^ Department of Rehabilitation Medicine, Qingtian People’s Hospital, Lishui, China, moh.gov.my; ^4^ Department of Rehabilitation Medicine, Changzhou Seventh People’s Hospital, Jiangsu Changzhou, China, moh.gov.my; ^5^ College of Pharmacy, Chonnam National University, Gwangju, South Korea, jnu.ac.kr

**Keywords:** anxiety, depression, kinesiophobia, low back pain, therapeutic aquatic exercise

## Abstract

**Objective:**

To examine whether kinesiophobia, anxiety, and depression mediate the effects of therapeutic aquatic exercise (TAE) versus physical therapy modalities (PTMs) on pain and disability in patients with chronic low back pain (CLBP).

**Methods:**

This study was a secondary analysis of data derived from a previously conducted randomized controlled trial, applying mediation analysis to explore potential psychological mediators. A total of 113 participants received 60‐min sessions of TAE or PTMs twice weekly for 3 months. The Roland–Morris Disability Questionnaire (RMDQ) and the numeric rating scale (NRS) were employed to measure the main outcome; the mediators included the Tampa Scale for Kinesiophobia (TSK), the Self‐Rating Anxiety Scale (SAS), and the Zung Self‐Rating Depression Scale (SDS). All mediation models reported causal pathway parameters, including intervention‐mediator and mediator‐outcome coefficients, with decomposition of total effect into direct and indirect effect (IE).

**Results:**

The mediation analysis found that kinesiophobia and depression mediated the effects of TAE compared with PTMs on the average pain (TSK [IE ‐0.203, 95% confidence interval, CI, −0.467 to −0.025]; SDS [IE −0.173, 95% CI −0.426 to −0.010]), the current pain (TSK [IE −0.151, 95% CI −0.371 to −0.013]; SDS [IE −0.220, 95% CI −0.495 to −0.030]), and the most severe pain (SDS [IE −0.186, 95% CI −0.476 to 0.004]) at 12 months. Meanwhile, kinesiophobia and depression mediated the TAE intervention effects on disability at 12 months (TSK [IE ‐0.174, 95% CI −2.176 to −0.270]; SDS [IE −0.555, 95% CI −1.470 to −0.014]). Anxiety mediated the effect of TAE on disability at 3 months (IE −0.365, 95% CI −0.832 to −0.065).

**Conclusion:**

Kinesiophobia, anxiety, and depression mediated noticeable improvements in pain and disability for patients with CLBP when conducting TAE intervention.

**Trial Registration:** Chinese Registry of Clinical Trial: ChiCTR1800016396

**Summary**
• Significant Statement
◦ TAE is a treatment option with long‐term therapeutic potential for CLBP patients, especially those with poor psychological states.

## 1. Introduction

Chronic low back pain (CLBP), a complex and multifactorial condition lasting over 12 weeks, is the leading global cause of disability and is projected to affect 800 million people by 2050 [1–3]. Rather than a symptom of a single disease, CLBP could be considered an individual’s protective mechanism in response to danger, threats, or homeostasis disruptions [4, 5], which is influenced by an interaction of physical, psychological, social, lifestyle, and genetic factors [6, 7]. Current guidelines consistently recommend exercise as first‐line therapy [8–10], with growing evidence supporting therapeutic aquatic exercise (TAE) as a safe and effective intervention [11–13]. TAE combines the benefits of exercise therapy with a water environment and provides different exercise intensities for patients with CLBP, even those with difficulty tolerating land‐based exercise therapy [14, 15]. In the last five years, several reviews on the effectiveness of TAE on CLBP supported that TAE can significantly reduce pain and dysfunctional levels in patients with CLBP [16, 17]. Nevertheless, the precise mechanism(s) by which TAE exerts its influence on CLBP outcomes remain elusive.

To our knowledge, pain level and physical function are predominantly listed as the main outcomes in CLBP intervention research [18, 19]. However, most exercise therapies do not directly affect pain and function, yet, may indirectly improve pain and function by enhancing muscle strength, altering biological force lines, and alleviating negative emotions [20]. Thus, understanding the mechanism through which specific exercise therapies occur is crucial to designing targeted and personalized treatment plans. Mediation analysis offers a quantified method to explore the relationships between the proposed mediator, outcomes, and intervening variables, which provides new insights to reveal the possible mechanisms of a certain intervention. Nowadays, studies on the mediating effect of CLBP have gathered much attention, with increasing evidence indicating that cognitive and psychological factors may act as potential mediators of pain and disability in individuals with CLBP [21–29]. A systematic review of mediation effects in chronic musculoskeletal pain revealed that most mediators associated with low back pain are psychological factors [21]. Some cross‐sectional studies reported that kinesiophobia, fear avoidance, depression, and stress mediate pain–disability associations in CLBP [22–24]. Several randomized controlled trials (RCTs) on LBP further reported the mediating roles played by kinesiophobia, fear avoidance, and depression during the intervention [25–29]. Two of these RCTs performed exercise therapy to improve pain and dysfunction in patients with CLBP; one revealed that pain catastrophizing and kinesiophobia mediated the effect of Pilates [27]; another reported the mediating role of pain catastrophizing during tai chi intervention [28].

While the therapeutic effects of TAE on CLBP are well‐documented, the underlying pathways mediating pain/functional improvements remain unexplored. Our previous RCT revealed that TAE showed more improvement in pain and disability compared to physical therapy modalities (PTMs) in patients with CLBP [13]. Based on that published paper, we conducted a secondary analysis to answer the mechanism‐involved question: To what degree are the differences in pain relief and functional improvement observed between TAE and PTMs mediated by the changes in kinesiophobia, anxiety, and depression?

## 2. Methods

### 2.1. Study Design

This is a secondary mediation analysis of a 3‐month intervention and 12‐month follow‐up RCT [13]. A total of 113 individuals with CLBP were recruited and randomly assigned to either the TAE group or the PTM group to receive the intervention twice a week for their 3 months and to complete follow‐up at 6 and 12 months. Only assessors in the experiment were blinded to the allocation and intervention program. All enrolled participants completed written informed consent before study initiation.

### 2.2. Interventions

The TAE group underwent a structured 60‐min aquatic exercise protocol consisting of a 10‐min warm‐up, 40‐min aquatic training, and a 10‐min recovery period.

The PTM group likewise received a total of 60 min of therapy, including 30 min of transcutaneous electrical nerve stimulation (TENS) and 30 min of infrared therapy. Both modalities were applied to the pain sites and delivered at an intensity that was comfortable for each participant [30, 31].

### 2.3. Data Collection

Outcome assessments included validated measures of CLBP‐related outcomes: numeric rating scale (NRS) for pain intensity and Roland–Morris Disability Questionnaire (RMDQ) for functional disability, respectively. The NRS evaluates pain on an 11‐point scale (0 = no pain and 10 = worst imaginable pain), capturing current, average, and worst pain over the past week [32]. The RMDQ consists of 24 yes/no items related to daily functioning, with total scores ranging from 0 to 24; higher scores indicate greater disability [33]. Both scales are widely used and validated in patients with CLBP.

The mediators included changes in scores from three self‐reported scales: the Self‐Rating Anxiety Scale (SAS), the Zung Self‐Rating Depression Scale (SDS), and the Tampa Scale for Kinesiophobia (TSK). Anxiety and depression were assessed using the SAS and SDS, respectively. Both scales consist of 20 items rated on a 4‐point scale, with total scores ranging from 20 to 80; higher scores indicate more severe symptoms [34, 35]. The TSK‐17 was used to assess fear of movement or re‐injury, with items rated on a 4‐point Likert scale. Total scores range from 17 to 68, with higher scores reflecting greater kinesiophobia. The TSK has shown good reliability and internal consistency in previous studies [36].

All outcome measures (NRS and RMDQ) and psychological mediators (SAS, SDS, and TSK) were assessed at four time points: baseline, postintervention (3 months), and at 6‐ and 12‐month follow‐ups. At each time point, one outcome variable was selected, and mediation analyses were conducted using the psychological mediators assessed concurrently.

### 2.4. Statistical Analysis

Mediation analyses were conducted using the multistep regression approach proposed by David A. Kenny [37]. Because all mediators and outcomes were continuous variables, ordinary least squares (OLS) multiple linear regression models were used to examine the relationships among the intervention (TAE vs. PTMs), psychological inventories (TSK/SAS/SDS), and clinical outcomes (NRS/RMDQ) [38, 39]. No additional covariates were included, as randomization was expected to balance baseline characteristics between groups. Model assumptions were evaluated through visual inspection of residual and Q–Q plots, and multicollinearity was assessed using variance inflation factors.

Taking the model in Figure 1(a) as an example, we applied a four‐step regression approach to test the mediation pathways:

1. Primary Regression: NRS (dependent variable) was regressed on the intervention variable (independent variables) to estimate the total effect (coefficient c) of the intervention on pain.
2. Mediation Analysis: TSK (mediator) was regressed on the intervention variable to quantify the effect of the intervention on the mediator (coefficient a).
3. Effect of Mediator on Outcome: NRS was then regressed on TSK to assess the association between the mediator and the outcome (coefficient b).
4. Combined Model: Finally, NRS was regressed simultaneously on both the intervention variable and TSK to estimate the direct effect of the intervention while accounting for mediation (coefficient c′). This step allows for the decomposition of the total effect (c) into the indirect effect (IE) (a∗b) and the direct effect (c′).

FIGURE 1The mediating role of TSK and SDS on the effect that TAE had on NRS average at 12‐month follow‐up. Models showing the mediating role of TSK on the relationship between TAE and NRS average at 12‐month follow‐up (a); the mediating role of SDS on the relationship between TAE and NRS average at 12‐month follow‐up (b), respectively. ^∗^: *p* < 0.05, ^∗∗^: *p* < 0.01, ^∗∗∗^: *p* < 0.001. The path coefficients are regression coefficients. Abbreviations: TAE, therapeutic aquatic exercise; NRS, numeric rating scale; TSK, Tampa Scale for Kinesiophobia; SDS, Zung Self‐Rating Depression Scale.

**Figure:**
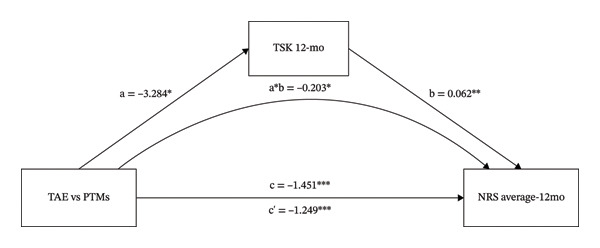
(a)

**Figure:**
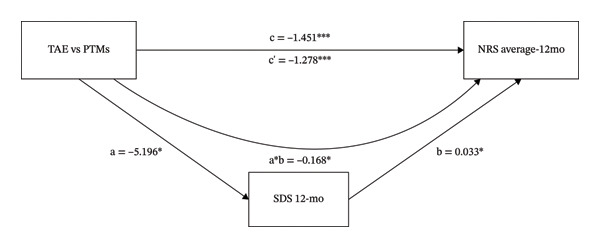
(b)

In this model, c′ represents the direct effect of the treatment on the outcome after accounting for the portion mediated by TSK. For instance, if TAE reduces fear of movement (TSK), which subsequently lowers pain (NRS), the product a∗b estimates this indirect pathway. The coefficient c′ captures the remaining effect of the treatment on NRS that is not explained by TSK. When both the IE (a∗b) and the direct effect (c′) are statistically significant, it supports a partial mediation model, indicating that TSK mediates part—but not all—of the treatment’s effect on pain. In contrast, if the IE is significant while the direct effect (c′) becomes nonsignificant, this suggests complete mediation, meaning the treatment influences the outcome primarily through its effect on TSK. The proportion mediated in Table 1 represents the value of a∗b/c, which indicates the proportion of the mediation effect. In addition, partially standardized IEs were reported as measures of effect size, along with their 95% confidence intervals (CIs).

**TABLE 1:** Direct and total effect coefficients and bootstrapping results (psychological factors as mediators).

Independent variable (X) intervention variable	Mediator (M)	Dependent variable (Y)	Coefficient a	Coefficient b	Coefficient c′ (direct effect)	Total effect c	Indirect effect (a^∗^b)		Bias‐corrected 95% CI		Proportion mediated	Partially standardized indirect effect	
							Point estimate	SE	Lower	Upper		Effect size	Bias‐corrected 95% CI
TAE vs. PTMs	TSK	NRS average (12 m)	−3.284^∗^	0.062^∗∗^	−1.249^∗∗∗^	−1.451^∗∗∗^	−0.203^∗^	0.114	−0.467	−0.025	0.140	−0.113	−0.248 to −0.014
TAE vs. PTMs	SDS	NRS average (12 m)	−5.196^∗^	0.033^∗^	−1.278^∗∗∗^	−1.451^∗∗∗^	−0.173^∗^	0.109	−0.426	−0.010	0.119	−0.096	−0.230 to −0.004
TAE vs. PTMs	TSK	NRS current (12 m)	−3.284^∗^	0.046^∗^	−1.208^∗∗∗^	−1.360^∗∗∗^	−0.151^∗^	0.093	−0.371	−0.013	0.111	−0.086	−0.204 to −0.008
TAE vs. PTMs	SDS	NRS current (12 m)	−5.196^∗^	0.042^∗∗^	−1.140^∗∗∗^	−1.360^∗∗∗^	−0.220^∗^	0.120	−0.495	−0.030	0.162	−0.125	−0.255 to −0.016
TAE vs. PTM	SDS	NRS most severe (12 m)	−5.196^∗^	0.036^∗^	−1.478^∗∗∗^	−1.664^∗∗∗^	−0.186^∗^	0.125	−0.476	0.004	0.112	−0.088	−0.217 to −0.0004
TAE vs. PTMs	TSK	RMDQ (12 m)	−3.284^∗^	0.358^∗∗∗^	−1.975^∗^	−3.149^∗∗^	−1.174^∗^	0.490	−2.176	−0.270	0.373	−0.204	−0.352 to −0.049
TAE vs. PTMs	SDS	RMDQ (12 m)	−5.196^∗^	0.107^∗^	−2.594^∗^	−3.149^∗∗^	−0.555^∗^	0.395	−1.470	0.014	0.176	−0.097	−0.250 to −0.005
TAE vs. PTMs	SAS	RMDQ (3 m)	−4.612^∗∗^	0.079^∗^	−1.035	−1.399^∗^	−0.365^∗^	0.201	−0.832	−0.065	0.261	−0.103	−0.224 to −0.019

*Note:* X, independent variable (intervention); M, mediator; Y, dependent variable (outcome); m, month; SDS, Zung Self‐Rating Depression Scale; SAS, Self‐Rating Anxiety Scale.

Abbreviations: CI, confidence interval; NRS, Numeric Rating Scale; PTMs, physical therapy modalities; RMDQ, Roland–Morris Disability Questionnaire; SE, standard error; TAE, therapeutic aquatic exercise; TSK, Tampa Scale for Kinesiophobia.

^∗^ *p* ≤ 0.05.

^∗∗^ *p* ≤ 0.01.

^∗∗∗^ *p* ≤ 0.001.

Preacher and Hayes’s bootstrap method [40] was applied to estimate IEs using 5000 percentile bootstrap samples, such as the different treatments potentially affecting TSK/SAS/SDS, which would influence NRS/RMDQ. Statistical significance was defined as 95% CIs excluding zero, indicating a significant mediated effect. All analyses followed the intention‐to‐treat principle. Statistical analyses were performed using SPSS 26.0, with *p* < 0.05 set as the significance threshold.

#### 2.4.1. Data Availability Statement

The data related to this study are not publicly accessible but may be obtained from the corresponding author upon reasonable request.

## 3. Results

The TAE group (*n* = 56) and the PTM group (*n* = 57) had a mean age of 31.72 ± 11.32 and 30.36 ± 11.76 years, respectively, with a mean duration of CLBP of 6.21 ± 5.65 and 7.28 ± 7.34 years. More detailed results of the RCT can be found in a previously published article [13]. Although mediation analyses were conducted for all time points and outcomes, only statistically significant findings were reported in the main text due to space constraints.

### 3.1. The Mediating Role of TSK and SDS on the Different Effects That TAE vs. PTMs had on NRS Average

As shown in Figure 1 and Table 1, the intervention variable and NRS averages at 12 months were significantly associated (β = −1.451, *p* < 0.001). In Figure 1(a), even after adjusting for TSK, the direct effect of the intervention variable on NRS averages remained significant (β = −1.249, *p* < 0.001). The intervention variable was also significantly associated with TSK (β = −3.284, *p* = 0.016), and TSK significantly predicted NRS averages (β = 0.062, *p* = 0.004). These results indicate that TSK might mediate the relationship between the intervention variable and the NRS average. In Figure 1(b), even after adjusting for SDS, the direct effect of the intervention variable on NRS averages remained significant (β = −1.278, *p* < 0.001). The intervention variable was also significantly associated with SDS (β = −5.196, *p* = 0.018), and SDS significantly predicted NRS averages (β = 0.033, *p* = 0.014). These results indicate that SDS might mediate the relationship between the intervention variable and NRS average. Bootstrap analyses (Table 1) showed that, after bias correction, the IEs of TSK and SDS were −0.203 (95% CI: −0.467 to −0.025) and −0.173 (95% CI: −0.426 to −0.010), respectively. As both 95% CIs excluded zero, these results indicate that TSK and SDS each partially mediated the relationship between the intervention variable and the NRS average score. Estimates of the partially standardized IEs and their bias‐corrected 95% CIs are provided in Table 1 as measures of effect size for all mediation models.

Table 1 presents the direct and total effect coefficients, bootstrapped IEs, and the corresponding partially standardized IEs with bias‐corrected 95% CIs as measures of effect size. For the effect of different treatments on the NRS average score, the proportion mediated was 0.140 for the TSK model and 0.119 for the SDS model, indicating that approximately 14.0% and 11.9% of the total effect may be explained by TSK and SDS, respectively.

### 3.2. The Mediating Role of TSK and SDS on the Different Effects That TAE vs. PTMs had on NRS Current

As shown in Figure 2 and Table 1, the intervention variable and NRS current at 12 months were significantly associated (β = −1.360, *p* < 0.001). In Figure 2(a), even after adjusting for TSK, the direct effect of the intervention variable on NRS current remained significant (β = −1.208, *p* < 0.001). The intervention variable was also significantly associated with TSK (β = −3.284, *p* = 0.016), and TSK significantly predicted NRS current (β = 0.046, *p* = 0.033). These results indicate that TSK might mediate the relationship between the intervention variable and NRS current. In Figure 2(b), even after adjusting for SDS, the direct effect of the intervention variable on NRS current remained significant (β = −1.140, *p* < 0.001). The intervention variable was also significantly associated with SDS (β = −5.196, *p* = 0.018), and SDS significantly predicted NRS current (β = 0.042, *p* = 0.001). These results indicate that SDS might mediate the relationship between the intervention variable and NRS current. Bootstrap analyses (Table 1) demonstrated that, after bias correction, the IEs of TSK and SDS were −0.151 (95% CI −0.371 to −0.013) and −0.220 (95% CI −0.495 to −0.030), respectively. As both 95% CIs excluded zero, these results indicate that TSK and SDS each partially mediated the relationship between the intervention variable and NRS current.

FIGURE 2The mediating role of TSK and SDS on the effect that TAE had on NRS current at 12‐month follow‐up. Models showing the mediating role of TSK on the relationship between TAE and NRS current at 12‐month follow‐up (a); the mediating role of SDS on the relationship between TAE and NRS current at 12‐month follow‐up (b), respectively. ^∗^: *p* < 0.05, ^∗∗^: *p* < 0.01, ^∗∗∗^: *p* < 0.001. The path coefficients are regression coefficients. Abbreviations: TAE, therapeutic aquatic exercise; NRS, Numeric Rating Scale; TSK, Tampa Scale for Kinesiophobia; SDS, Zung Self‐Rating Depression Scale.

**Figure:**
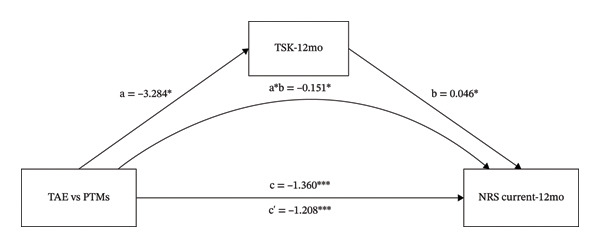
(a)

**Figure:**
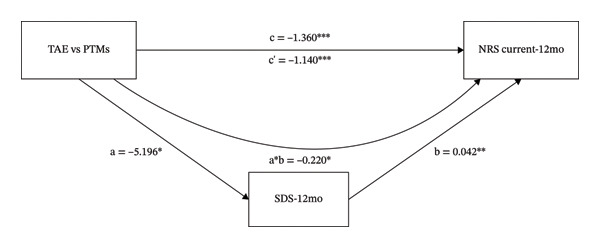
(b)

As shown in Table 1, the proportion mediated was 0.111 for the TSK model and 0.162 for the SDS model, suggesting that approximately 11.1% and 16.2% of the total effect may be explained by TSK and SDS, respectively.

### 3.3. The Mediating Role of SDS on the Different Effects That TAE vs. PTMs had on NRS Most Severe

**FIGURE 3:**
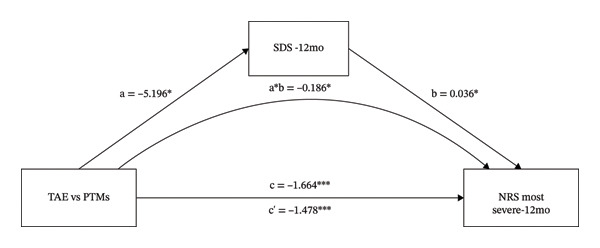
The mediating role of SDS on the effect that TAE had on NRS most severe at 12‐month follow‐up. ^∗^: *p* < 0.05, ^∗∗^: *p* < 0.01, ^∗∗∗^: *p* < 0.001. The path coefficients are regression coefficients. Abbreviations: TAE, therapeutic aquatic exercise; NRS, Numeric Rating Scale; SDS, Zung Self‐Rating Depression Scale.

As shown in Figure 3 and Table 1, the intervention variable and NRS most severe at 12 months were significantly associated (β = −1.664, *p* < 0.001). Even after adjusting for SDS, the direct effect of the intervention variable on NRS most severe remained significant (β = −1.478, *p* < 0.001). The intervention variable was also significantly associated with SDS (β = −5.196, *p* = 0.018), and SDS significantly predicted NRS most severe (β = 0.036, *p* = 0.026). These results indicate that SDS might mediate the relationship between the intervention variable and NRS most severe. Bootstrap analysis (Table 1) showed that, after bias correction, the IE of SDS was −0.186 (95% CI: −0.476 to 0.004). As the CI did not include zero, this suggests that SDS partially mediated the relationship between the intervention variable and NRS current.

As shown in Table 1, the proportion mediated was 0.112 for the SDS model, suggesting that approximately 11.2% of the total effect may be explained by the SDS.

### 3.4. The Mediating Role of TSK, SDS, and SAS on the Different Effects That TAE vs. PTMs had on RMDQ

As shown in Figure 4(a), the intervention variable and RMDQ at 12 months were significantly associated (β = −3.149, *p* = 0.003). In Figure 4(a), even after adjusting for TSK, the direct effect of the intervention variable on RMDQ remained significant (β = −1.975, *p* = 0.041). The intervention variable was also significantly associated with TSK (β = −3.284, *p* = 0.016), and TSK significantly predicted RMDQ (β = 0.358, *p* < 0.001). These results indicate that TSK might mediate the relationship between the intervention variable and RMDQ. In Figure 4(b), even after adjusting for SDS, the direct effect of the intervention variable on RMDQ remained significant (β = −2.594, *p* = 0.015). The intervention variable was also significantly associated with TSK (β = −5.196, *p* = 0.018), and SDS significantly predicted RMDQ (β = 0.107, *p* = 0.019). These results indicate that SDS might mediate the relationship between the intervention variable and RMDQ. Bootstrap analyses (Table 1) showed that, after bias correction, the IEs of TSK and SDS were −0.174 (95% CI −2.176 to −0.270) and −0.555 (95% CI −1.470 to −0.014), respectively. As both CIs excluded zero, these results indicate that TSK and SDS each partially mediated the relationship between the intervention variable and RMDQ.

FIGURE 4The mediating role of TSK, SDS, and SAS on the effect that TAE had on RMDQ. Models showing the mediating role of TSK on the relationship between TAE and RMDQ at 12‐month follow‐up (a); the mediating role of SDS on the relationship between TAE and RMDQ at 12‐month follow‐up (b); and the mediating role of SAS on the relationship between TAE and RMDQ at 3‐month follow‐up (c), respectively. ^∗^: *p* < 0.05, ^∗∗^: *p*  <  0.01, ^∗∗∗^: *p* < 0.001. The path coefficients are regression coefficients. Abbreviations: TAE, therapeutic aquatic exercise; TSK, Tampa Scale for Kinesiophobia; RMDQ, Roland–Morris Disability Questionnaire; SAS, Self‐Rating Anxiety Scale.

**Figure:**
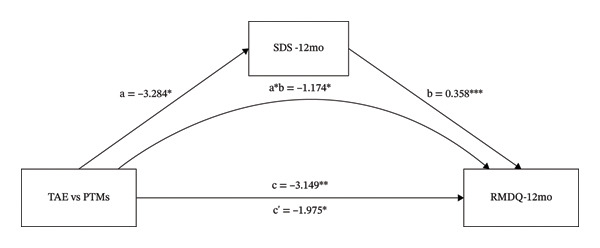
(a)

**Figure:**
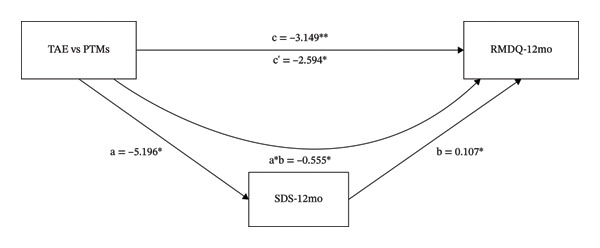
(b)

**Figure:**
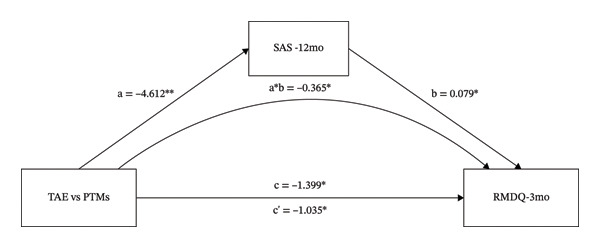
(c)

As shown in Figure 4(c), the intervention variable and RMDQ at 3 months were significantly associated (β = −1.399, *p* = 0.0352). Adjusting for SAS eliminated the significance of the direct effect of the intervention variable on RMDQ (β = −1.035, *p* = 0.123), which is consistent with a full mediation model. The intervention variable was significantly associated with SAS (β = −4.612, *p* = 0.009), and SAS significantly predicted RMDQ (β = 0.079, *p* = 0.028). These results indicate that SAS might mediate the relationship between the intervention variable and RMDQ. Bootstrap analysis (Table 1) showed that, after bias correction, the IE of SAS was −0.365 (95% CI −0.832 to −0.065). As the CI did not include zero, this suggests that SAS mediated the relationship between the intervention variable and RMDQ.

As shown in Table 1, the proportion mediated was 0.373, 0.176, and 0.261 for the TSK, SDS, and SAS models, suggesting that approximately 37.3%, 17.6%, and 26.1% of the total effect may be explained by changes in kinesiophobia, depression, and anxiety, respectively.

### 3.5. Subgroup Mediation and Reverse Mediation Analyses

Supporting Table 1 divides the participants in the TAE group into groups with or without anxiety (SAS ≥ 50 vs < 50) and with or without depression (SDS ≥ 53 vs < 53) and examines group differences in NRS and RMDQ scores at different time points. Further mediation analysis was conducted on items with significant differences and only found that SDS mediated the distinct impacts of TAE on RMDQ at 3 months across subgroups with or without anxiety. As shown in Supporting Figure 1, subgroups with or without anxiety significantly predicted RMDQ at 3 months (β = −2.354, *p* = 0.033). However, after adjusting for SAS, the direct effect between the subgroup and the RMDQ lost significance (β = −0.490, *p* = 0.674), supporting full mediation. Meanwhile, the subgroup was significantly associated with SDS (β = −13.896, *p*< 0.001), and SDS significantly predicted RMDQ (β = 0.134, *p* = 0.003). Bootstrap analysis further confirmed the mediating role of SDS, with a bias‐corrected IE of −1.864 (95% CI: −3.390 to −0.593). These findings indicate that SDS mediated the relationship between anxiety subgroup status and disability.

Supporting Table 2 shows the mediating roles of pain intensity in the effects of the intervention on TSK and SDS. Supporting Figure 2 illustrates the mediating roles of NRS average and NRS current in the effect of TAE on TSK at the 12‐month follow‐up. Supporting Figure 3 shows the mediating roles of NRS average, NRS current, and NRS most severe in the effect of TAE on SDS at the 12‐month follow‐up.

## 4. Discussion

This study investigated the mediating effect of psychological indicators on reducing pain and improving function in patients with CLBP when TAE was compared with PTMs, to determine the possible mechanisms through which TAE works on CLBP. An earlier study showed that TAE was statistically and clinically superior to PTMs in improving pain and disability [13]. Compared with the PTM group, the TAE group showed greater improvements in the pain and disability after adjusting for sex, age, body mass index, physical activity, and so on. Our findings further indicate that changes in kinesiophobia, anxiety, and depression affect pain and dysfunction outcomes between two groups, with most effects observed at the 12‐month follow‐up.

For many people with CLBP, kinesiophobia acts as an initial protective mechanism to prevent the potential deterioration of their condition [41], but persistent kinesiophobia can lead to long‐lasting CLBP, which is even more disabling than the pain itself [42]. Physical exercise was the most commonly used method to treat an irrational fear of movement [43]. In our prior RCT, it was demonstrated that TAE exerted a beneficial influence on kinesiophobia among patients with CLBP when compared to PTMs [13]. One RCT showed that, for patients with chronic spinal pain, kinesiophobia mediated the improvement in physical function and quality of life, and reductions in medication use [44]. Interestingly, in this study, the mediating effects of the changes in kinesiophobia on both pain and functioning were observed only at the12‐month follow‐up, with no significant mediation at 3 or 6 months. One possible explanation is that some patients with CLBP may have been afraid of exercise and potentially even more hesitant to exercise in an unfamiliar aquatic environment. These individuals might have continued to experience a “fear‐avoidance” cycle while engaging in TAE, which could have limited the immediate impact of the intervention on reducing fear of movement during the early stages of treatment. After completing the program and experiencing sustained pain relief, they may have gradually regained confidence to participate in activities, thereby reducing the adverse effects of kinesiophobia. However, as the included study assessed kinesiophobia using the TSK but did not measure treatment expectancy, aquatic‐specific fear, or self‐efficacy, this interpretation should be considered speculative and hypothesis‐generating rather than confirmatory.

Pain often triggers negative emotions, such as anxiety and depression, which in turn exacerbate pain, creating a self‐reinforcing cycle [45]. One study found that depression and stress accounted for about 30% of the pain‐disability link in subacute LBP [23]. TAE has a positive effect on reducing depression and anxiety [46]. In our study, anxiety mediated group differences in functional improvement at 3 months, while depression mediated pain reduction at follow‐up. Notably, SDS played a mediating role in the effect of TAE on RMDQ at 3 months between the anxiety and nonanxiety groups. Anxiety and depression are two of the most frequently mentioned terms when referring to psychological problems. Anxiety often reflects anticipatory fear, whereas depression involves prolonged negative affect. The engaging nature of TAE may have distracted patients from pain‐related vigilance and potentially reduced anxiety. The group‐based format might also have provided opportunities for social interaction and perceived support, which could have helped address pain‐related misbeliefs and foster confidence in daily activities, thereby possibly contributing to functional improvements. However, as constructs, such as social support, pain beliefs, and self‐efficacy, were not directly assessed in the included studies, these interpretations should be considered speculative and hypothesis‐generating rather than confirmatory. In contrast, depression, as a more persistent psychological state, appeared less responsive in the short term. Pain relief during follow‐up may have gradually alleviated depressive symptoms, though mediation analysis at 12 months showed a marginal IE, suggesting a modest long‐term impact of TAE on depression.

From a temporal perspective, anxiety was found to mediate the positive effects of aquatic exercise on functional improvement at the end of the 3‐month intervention. By the 12‐month follow‐up, the benefits of aquatic exercise on both pain and dysfunction were partially mediated by kinesiophobia and depression. This time‐dependent pattern may be attributed to the differing nature and responsiveness of anxiety, depression, and kinesiophobia. Anxiety typically reflects heightened sensitivity and anticipation of pain, which can be more immediately influenced by short‐term interventions. In contrast, depression is often a more persistent and internalized emotional state, while kinesiophobia is usually accompanied by avoidance behaviors—both of which may require longer periods of sustained symptom relief to show meaningful improvement. These findings underscore the importance of considering the temporal dynamics of psychological mediators in the treatment of CLBP and suggest that different psychological mechanisms may contribute to outcomes at different stages of recovery. Interestingly, the results suggest that SAS fully mediated the relationship between the intervention and RMDQ at 3 months. This does not imply a lack of treatment efficacy; rather, it highlights anxiety as a key psychological mechanism underlying the short‐term therapeutic effect.

The potential mechanisms of many therapies for CLBP have been proven to be psychological [21]. A recent systematic review suggested that a strong fear of movement at baseline in patients with LBP affects pain intensity at the 6‐month follow‐up after treatment [47]. Lianne [27] performed a secondary analysis of an RCT and found that compared to controls, pain catastrophe mediated 20%–34% of Pilates effects on pain level and physical function, while kinesiophobia accounted for 55% of Pilates‐related functional improvements. Hall [28] designed an RCT employing 10‐week tai chi vs. wait‐list control for LBP and reported that about one‐third of the changes in pain level and bothersomeness and two‐thirds of the improvements in dysfunction could be attributed to the mediating effect of catastrophizing. Our mediation analysis suggested that the greater improvements in pain observed in the TAE group compared with the PTM group over the 12‐month follow‐up were partially explained by reductions in kinesiophobia and depression. Specifically, approximately 11.1%–14.0% of the total effect was mediated by reductions in kinesiophobia and 11.2%–16.2% by improvements in depression. Similarly, the superior improvements in physical function in the TAE group were partially explained by changes in psychological factors. Approximately 26.1% of the total effect after the three‐month intervention was associated with reductions in anxiety, while 37.3% and 17.6% of the effect during the 12‐month follow‐up were associated with reductions in kinesiophobia and depression, respectively. These data are lower than the proportion of mediating effects exerted by psychological factors reported in the two previous studies. We hypothesize the reason may be that most of the participants in this study were relatively young with lower levels of kinesiophobia, anxiety, and depression.

Although guidelines recommend exercise therapy as the first‐line treatment for patients with CLBP, in practice, most doctors and patients opt for passive forms of treatment [48]. The effects of exercise therapy are not as expected, and psychological factors may be a major barrier to its effectiveness. These findings may enable clinicians to optimize TAE regimens biopsychosocially by addressing psychosocial factors directly. First, clinical workers need to do a biopsychosocial assessment of patients with CLBP to improve their understanding of biopsychosocial performance [49]. Secondly, clinicians could improve the experience of participating in TAE for patients with CLBP by providing better supervised or structured group exercise to alleviate fear of movement and concerns regarding pain. While developing exercise prescriptions, information about the benefits of TAE, exercise precautions, and other information should be provided to patients with CLBP through verbal and nonverbal communication to enable them to recognize the importance and safety of TAE and have enough confidence to engage in it.

### 4.1. Strength

This appears to be the first study examining the mediating factors that influence the efficiency of TAE in improving pain and dysfunction for CLBP, allowing researchers to clarify the possible mechanisms of TAE and providing preliminary evidence to inform future exercise program design. Secondly, this mediator study is a secondary analysis using data from a high‐quality RCT, and the original trial has the advantages of large sample, concealed allocation, intention‐to‐treat analysis, high data completeness, and reliable measurements, which methodologically guarantees the study data quality. In addition, using regression analyses and including data from multiple follow‐up measurements in this study allowed us to draw stronger conclusions than baseline or postintervention measurements analyzed as mediators.

### 4.2. Limitations

This study has several limitations. Firstly, as a secondary analysis of an RCT, the original data were not specifically tailored to the aims of this mediation study. The trial was primarily designed to evaluate pain and functional outcomes, and the two intervention modalities differed substantially (active TAE vs. passive PTMs), which may influence the interpretation of the mediation effects. In addition, unmeasured psychological variables—such as fatigue and self‐efficacy—may also mediate the effects of TAE in CLBP. Thus, the proposed mechanisms may be incomplete and should be used cautiously. However, rigorous mediation analyses were conducted for each psychological factor separately across different follow‐up time points to ensure the robustness of the findings. Secondly, although we used the Baron and Kenny regression framework combined with Preacher and Hayes’s bootstrap approach to test mediation effects, residual confounding between mediators and outcomes may bias results, and the statistical limitations of classical mediation models should be acknowledged. Additionally, the sample consisted predominantly of young adults, limiting the generalizability of the findings to other populations. Moreover, subgroup analyses by anxiety and depression status were exploratory due to small sample sizes. Finally, postintervention adherence and additional treatments were not tracked, which may influence long‐term outcomes. Future studies should include larger, more diverse cohorts and consider qualitative methods to explore how TAE influences lifestyle and self‐management in CLBP and then provide a basis for explaining the long‐term effects of TAE.

## 5. Conclusion

Consistent with previous RCT evidence showing that TAE is superior to PTMs in improving pain and functional outcomes in patients with CLBP, the present mediation analysis suggests that these benefits may be partially explained by reductions in kinesiophobia, anxiety, and depression, with each psychological factor influencing outcomes at different follow‐up stages. Notably, most mediating effects were observed only at the 12‐month follow‐up, indicating that TAE may have sustained positive effects on psychological status, which in turn contribute to long‐term improvements in pain and functional disability. Together, these findings support the potential long‐term value of TAE, particularly for patients with adverse psychological profiles.

## Author Contributions

This study was designed by Mengsi Peng and Yanan Zheng. The experiments were performed by Changcheng Chen, Juan Wang, Shengfeng Liu, and Mengsi Peng. The data were analyzed by Mengsi Peng and Yanan Zheng, and the results were critically examined by all authors. Shengfeng Liu had a primary role in preparing the manuscript, which was edited by Mengsi Peng and Yanan Zheng.

## Funding

This work was supported by the Joint Program on Health Science & Technology Innovation of Hainan Province (No: WSJK2024QN114).

## Disclosure

All authors have approved the final version of the manuscript and agreed to be accountable for all aspects of the work.

## Ethics Statement

This study obtained ethical clearance from the Institutional Review Board (IRB) at Shanghai University of Sport (No. 2018042).

## Consent

All subjects were informed of the experiment and signed informed consent before participating in the experiment.

## Conflicts of Interest

The authors declare no conflicts of interest.

## Supporting Information

Additional supporting information can be found online in the Supporting Information section.

Supporting Description.

## Supporting information


**Supporting Information 1** Supporting File 1. Study protocol.

**Supporting Information 2** Supporting Figure 1. The mediating role of SDS on the effect that TAE had on RMDQ at 3‐month for subgroup with anxiety. ^∗^: *p* < 0.05, ^∗∗^: *p* < 0.01, ^∗∗∗^: *p* < 0.001. The path coefficients are regression coefficients. Abbreviations: TAE, therapeutic aquatic exercise; SDS, Zung Self‐Rating Depression Scale; RMDQ, Roland‐Morris Disability Questionnaire.

**Supporting Information 3** Supporting Figure 2. The mediating role of NRS average and NRS current on the effect that TAE had on TSK. Models showing the mediating role of NRS average on the relationship between TAE and TSK at 12‐month follow up (a); and the mediating role of NRS current on the relationship between TAE and TSK at 12‐month follow up (b); respectively. ^∗^: *p* < 0.05, ^∗∗^: *p* < 0.01, ^∗∗∗^: *p* < 0.001. The path coefficients are regression coefficients. Abbreviations: TAE, therapeutic aquatic exercise; NRS, Numeric Rating Scale; TSK, Tampa Scale for Kinesiophobia.

**Supporting Information 4** Supporting Figure 3. The mediating role of NRS average, NRS current and NRS most severe on the effect that TAE had on SDS at 12‐month follow up. Models showing the mediating role of NRS average on the relationship between TAE and SDS at 12‐month follow up (a); the mediating role of NRS current on the relationship between TAE and SDS at 12‐month follow up (b); and the mediating role of NRS slightest on the relationship between TAE and TSK at 12‐month follow up (c); respectively. ^∗^: *p* < 0.05, ^∗∗^: *p* < 0.01, ^∗∗∗^: *p* < 0.001. The path coefficients are regression coefficients. Abbreviations: TAE, therapeutic aquatic exercise; NRS, Numeric Rating Scale; SDS, Zung Self‐Rating Depression Scale.

**Supporting Information 5** Supporting Table 1. Subgroup analysis of the therapeutic effect of TAE on pain and dysfunction in patients with CLBP. Abbreviations: TAE, therapeutic aquatic exercise; CLBP, chronic low back pain; CI, confidence interval; NRS, Numeric Rating Scale; m, month; RMDQ, Roland‐Morris Disability Questionnaire.

**Supporting Information 6** Supporting Table 2. Direct and total effect coefficients and the results of bootstrapping (pain intensity as mediators). Abbreviations: X, independent variable (intervention variable); M = mediator; Y, dependent variable (outcome); CI, confidence interval; SE, standard error; TAE, therapeutic aquatic exercise; PTMs, physical therapy modalities; NRS, Numeric Rating Scale; m, month; TSK, Tampa Scale for Kinesiophobia; SDS, Zung Self‐Rating Depression Scale; SAS, Self‐Rating Anxiety Scale. ^∗^ *p* ≤ 0.05; ^∗∗^ *p* ≤ 0.01; ^∗∗∗^ *p* ≤ 0.001.

## Data Availability

The data are available from the corresponding author upon reasonable request.
