# Supplementary material for: Effects of Therapeutic Aquatic Exercise Versus Physical Therapy Modalities on Pain and Disability in People With Chronic Low Back Pain: Potential Mediating Roles of Kinesiophobia, Anxiety, and Depression
Source: Pain Res Manag. 2026 Apr 12;2026:5537314. doi: 10.1155/prm/5537314 (PMC13071334; doi:10.1155/prm/5537314)
Supplement: Supplementary file 1 — Supporting Information 1 Supporting File 1. Study protocol. [file PRM-2026-5537314-s005.docx]

**Supplementary file 1 study protocol**

Effects of 12-Week Therapeutic Aquatic Exercise for Patients with Chronic Low Back Pain: Protocol of a Single-blind Randomized Controlled Trial

**Introduction**

Chronic low back pain (CLBP), a symptom rather than a disease, is commonly located between the lower rib and the buttocks wrinkles.^1^ Similar to other symptoms, CLBP results from variable factors, and the specific underlying cause can be rarely identified.^2^ People of all ages may suffer from CLBP, it is uncommon in the first decade of life; however, the prevalence increases steadily during adolescence and peaks in midlife.^1^ The lifetime morbidity of CLBP worldwide is up to 84%.^3^ CLBP was once seen as a short-term condition but is now considered as a long-term condition because of its frequent recurrent episodes. Approximately 33% of people experience relapse within 1 year after recovery.^1,3^ Given its high prevalence and chronicity, CLBP is the leading cause of disability across the world, causing more disabilities than any other chronic diseases do.^1,4,5^ From 2013 to 2016, CLBP consistently remained the top six most costly global health issues.^6-8^ Consultation about back pain accounts for 7% of all general practitioner consultations,^3^ and people with CLBP miss 4.1 million working days in 1 year.^9^ Undoubtedly, low back pain is now a substantial public health challenge worldwide.

Drug therapy is one of the most commonly used treatments for CLBP, but side effects due to medication are inevitable.^10,11^ Considering that the condition of most patients with CLBP improves naturally regardless of treatment, nonpharmacologic and nonsurgical methods should be the first-line choice.^9,12^ Clinical applications in patients with CLBP, physical therapy modalities (PTMs), such as transcutaneous electrical nerve stimulation (TENS) and far-infrared (FIR) irradiation therapy, is regard as a relatively safe, convenience and non-invasive option.^13,14^ Nowadays, exercise therapy is widely recommended as a valid way for pain relief and functional improvements by most clinical guidelines.^3-5,9^ Among the numerous exercise therapies, therapeutic aquatic exercise (TAE) is often prescribed by clinical doctors and is becoming increasingly popular for alleviating pain and facilitating function.^15,16^

TAE refers to water-based treatments or exercise. With various properties, including its buoyancy pressure, density, thermal capacity, and conductivity,^15^ water is an ideal environment to conduct an exercise program. TAE reduces the stress on intervertebral disk and intervertebral joint with the help of hydrostatic buoyancy; enables a large range of movement by supporting the body weight; improves lumbar muscle tone via its natural resistance; offers gentle manipulation on back because of turbulence and wave propagation; adjusts the velocity of exercise by changing the depth of water; moreover, dynamic water environment improves microcirculation, enhances balance and coordination, facilitates relaxation, and decreases the contracture.^16-20^

Some studies have shown that TAE aquatic exercise can be a safe and effective treatment approach for CLBP.^20-23^ Whether combined with other therapies or used alone, TAE is both beneficial to pain reduction and functional improvement.^24,25^ Although the effect of aquatic therapy on patients with CLBP has been explored,^25,26^ the duration of most previous studies was short and did not include a long follow-up period. Both as a kind of non-invasive treatment, there are no studies investigating whether TAE could produce more benefits than PTMs for people with CLBP. Therefore, we will perform a single-blind randomized controlled trial (RCT) with long follow-up to observe the effects of TAE on people with CLBP versus PTMs.

**Methods/Design**

**Research goals and hypotheses**

We will design an RCT to conclude TAE and PTMs for people with low back pain to see:

Goal 1: whether TAE compared favorably with PTM for people with CLBP;

hypotheses for Aim 1: Patients in TAE group will receive more benefits in pain relieve and functional improvement than subjects in the PTMs group. There will be significant differences between two groups.

Goal 2: whether TAE yields long-term effects.

hypotheses for Aim 2: TAE will create a long-term effect on people with CLBP while PTMs will not. There will be significant differences between two groups during the fellow-up period.

**Study design**

This study is a 3-month single-blind RCT with a 12-month follow-up (Trial registration: ChiCTR1800016396). All included participants will be randomly allocated into either the TAE group or the PTMs group. The interventions of two groups will be carried out in Shangti indoor constant temperature swimming pool and Shanghai Shangti Orthopeadics Hospital respectively. Experienced physiotherapists will carry out the measurements at baseline, after the 3-month intervention, at 6-month follow-up, and at 12-month follow-up from the beginning to determine whether short-term effects and long-term impacts will be achieved.

**Participant selection**

Subjects who meet all the inclusion criteria and do not have any of the exclusion criteria will be eligible for enrollment. Before undergoing other tests, participants will be examined via a questionnaire with the following entries: basic information (e.g., age, sex, height and weight), physical activity, medical history, medical expenditure, back pain duration, back pain intensity (at present, over the last week, the worst pain, and the average pain), and self-rated back function.

**Inclusion criteria are as follows:**

1. Aged ranged from 18 to 65; 2. Pain, muscle tension or stiffness between the buttock band and the rib arch; may also have lower limb pain; 3. Pain intensity (when the most painful) ≥ 3 on the numeric rating scale (NRS); 4. CLBP lasting at least 3 months; 5. Voluntary participation in the trial and with written informed consent; 6. Accepted randomization. Subjects will be included if they meet all criteria above.

**Exclusion criteria are as follows:**

1. mental illness or cognitive impairment (Mini-mental State Examination, <24); 2. Specific lumbago, such as fracture, spinal stenosis, tumour infection and spinal structural abnormalities; 3. Lumbar dysfunction or pain caused by other diseases; 4. Complex back problems, such as spinal surgery; 5. Patients with severe and unstable cardiovascular, renal or liver diseases; 6. Received a regular low back pain exercise intervention during the past 6 months; 7. Pregnant or lactating; 8. Chlorine allergy; 9. Water-related anxiety or unable to adapt to an aquatic environment; 10. Urinary or faecalis incontinence; 11. Contagious skin diseases, ulcers or open wounds; 12. Medications that alter sensory perception. Subjects will be excluded if they have any of these criteria.

**Withdrawal criteria**

1. Patients lose interest; 2. Patients’ schedule conflict with the experimental arrangement; 3. Patients develop serious conditions, such as stroke; 4. Patients experience a side effect due to TAE or PTMs.

**Ethical considerations**

Before the measurement will implemented, informed consent will be signed by all participants interested in the study. The project is ratified by the ethics committee of the Shanghai University of Sport (number 2018042), Shanghai, China.

**Randomization and blinding**

Randomization in a 1:1 ratio will be performed according to a computer-generated scheme, and participants will be assigned into the control group (PTMs) or the experimental group (TAE). A researcher who carries out the randomization with sealed and opaque envelopes will remain separated from the intervention team.

A group of assessors will be responsible for each block of the measurement, but they are not aware of the group assignments and remain distant from the invention. The instructors are masked to the study’s hypothesis and experimental purpose. To be ignorant of grouping when receiving a behavior-related treatment at the same time is impossible for participants.

**Interventions**

The intervention sessions will be carried out by a group of qualified physicotherapists who will not take part in the data collection. Both programs will last 12 weeks and will be administered twice per week for a total of 24 treatment sessions. The participants will be encouraged to complete the intervention as designed, and the adherence rate is expected to be at least 75%. Attendance frequency, medication changes, and adverse events during the sessions and after treatment will be filled out in a daily record form. Once a participant absents from the intervention sessions, he or she will be contacted immediately to determine the reason. Participants who withdraw halfway, fail to attend the evaluations, or miss more than 2 weeks will be regarded as drop-outs ^27^.

**TAE group**

The temperature will be customized at 30 °C of water and 27.5 °C of environment to elicit the same heart response both in water and in air. The entire intervention will be completed in a swimming pool with a dimension of 20 m × 6 m and a depth of 1.3–1.5 m. To ensure that all of the participants will be submerged at their xiphoid bone level, some aquatic steps will be placed underwater as auxiliary forces.

The TAE protocol will be designed by the researcher in accordance with available scientific evidence. During the exercise, the participants will receive verbal, tactile, and visual information to correct their movements and ensure that the lumbar spine remains in a neutral position while standing. Thus, excessive loading on the spine will be avoided, and trunk muscles will be activated. The target exercise intensity will depend on the subject’s self-rated score of Rating of Perceived Exertion of approximately 13 in accordance with 60%–80% of maximum heart rate.^28^

The participants will start the exercise with an active warm-up for 10min to enhance neuromuscular activation. In the succeeding 40 min, they will perform an aquatic session, including abdominal bracing, vertical downward press, lateral downward press, slant downward press, straight leg raising, treading water, and deep water running. Finally, the participants will have a cool-down period for 10min. Table 1 shows the full description of the protocol.

All subjects will participate in the exercise in a group of 8–9 people. Weeks 1 and 2 are the learning period for subjects to become familiar with the actions. Then, the intensity of movements will be adjusted by changing the kickboard sizes. Thus, the neuromuscular stimulation will be maximized, and the subjects’ interest will also be maintained.

**PTMs group**

The subjects in the control group received TENS and IR irradiation therapy. Both modalities were focused on pain points, and each had a duration of 30 min.

TENS (model KD-2A, Beijing Yiyang Kangda Medical Instrument Co., Ltd., China) sends a bidirectional asymmetrical square wave at a frequency of 2 Hz to 160 Hz and a pulse width of 20 µs to 500 µs. The participants received TENS at a current frequency of 120 Hz and a pulse width of 100 µs. Disposable surface electrodes with dimensions of 50 mm × 50 mm were placed on the pain point, and pulse intensity was adjusted to produce a comfortable tingling sensation.^29,30^

The IR apparatus (model LY-607A Foshan Lingyuan Medical Technology Co., Ltd., China) was placed with its lamp located 50 cm to 75 cm above the exposed area. The lamp direction was adjusted to ensure that radiation struck the surface at or near a right angle such that maximum penetration was achieved.^31,32^ At 3–5 min after the instrument was energised, the patient was asked whether their sense of warmth was appropriate, and the lamp height was adjusted to prevent scalding.

**Outcome measures**

Clinical outcomes are classified as primary outcomes and secondary outcomes, including pain intensity, function of low back pain, and adverse events.

**Primary outcomes**

Disability was assessed with the Roland Morris Disability Questionnaire (RMDQ), which contains 24 items that are closely related to the daily life activities of patients with CLBP.^34^ The scores are as follows: 1 for checking ‘YES’ and 0 for checking ‘NO’. The final score varies from 0 to 24. Higher scores are associated with more severe disability.^35^

**Secondary outcomes**

1. Pain intensity was measured with the NRS, which consisted of 11 numbers from 0 to 10. The scores are set as follows: 0 as painless, 1–3 as mild pain, 4–6 as moderate pain and 7–10 as strong and unbearable pain. The subjects reported the pain intensity they feel at the present and that they experienced last week (slightest, average and most serious).^33^

2. Quality of life was measured with the Short-form (36) Health Survey (SF-36). SF-36 consists of eight scales, and a high score indicates low disability. The SF-36 questionnaire is reliable and relatively stable considering that its overall Cronbach’s α coefficient = 0.791 and r = 0.778.^36^

3. Anxiety state was measured with the self-rating anxiety scale (SAS). SAS is a 20-item self-reported assessment device.^37^ Each question is based on the following responses: ‘rarely’, ‘sometimes’, ‘usually’ and ‘most of the time.’ A respondent should choose the appropriate statement on the basis of his condition within the past 1 or 2 weeks. The total raw score may vary from 20 to 80, a high score indicates high anxiety levels.^37^

4. Depression state was measured with the Zung Self-Rating Depression Scale (SDS). The SDS is a 20-item self-reported questionnaire covering affective, psychological and somatic symptoms associated with depression. Each item is scored on a Likert scale that ranges from 1 to 4. Total scores range from 20 to 80, wherein 20–44 is normal, 45–59 is mildly depressed, 60–69 is moderately depressed and >70 is severely depressed.

5. Sleep quality was measured with the Pittsburgh Sleep Quality Index (PSQI). The PSQI is a self-reported scale that is used to assess sleep quality over a 1-month period. The PSQI consists of 19 individual items and comprises 7 components that are used evaluate sleep quality from several different aspects, such as sleep latency, sleep duration, habitual sleep efficiency, sleep disturbances, sleeping medication use and daytime dysfunction. The global score of PSQI ranges from 0 to 21, wherein high values indicate poor sleep quality.^38^

6. The Pain Anxiety Symptoms Scale (PASS) was used to evaluate pain-induced avoidance, fear, cognitive anxiety and physiological anxiety. The PASS contains 20 items. Each item is divided into 6 grades: ‘never’, ‘occasionally’, ‘sometimes’, ‘often’, ‘almost always’ and ‘always’. The total score ranges from 0 to 100. High scores are indicative of severe pain and anxiety symptoms. The internal consistency of the scale is good with Cronbach α = 0.92 and test–retest reliability intraclass correlation coefficient (ICC) = 0.90.^39^

7. Kinaesiophobia was measured with the Tampa Scale for Kinaesiophobia (TSK). The 17-item version of the TSK is used to assess the fear of activity or (re)injury resulting from pain.^40^ It is rated by using a 4-point Likert scale that varies from ‘strongly disagree’ to ‘strongly agree’. The total score of this scale varies from 17 to 68, wherein a high score reflects high levels of kinaesthetic phobia.^40^ The TSK has been confirmed to have acceptable reliability (ICC = 0.86) and excellent validity (Cronbach’s α = 0.74).^41^

8. The Fear Avoidance Beliefs Questionnaire (FABQ) was used to evaluate fear avoidance belief. The FABQ consists of 16 items, including 2 subscales that are used to evaluate the effects of fear avoidance beliefs on physical activity (items 1–5) and work (items 6–16). Each item is scored from 0 to 6. These scores correspond to strongly disagree, very disagree, disagree, uncertainty, agree, very agree and strongly agree. The total score of this scale ranges from 0 to 96. High scores indicate strong fear avoidance belief.

9. Minimal clinically important difference (MCID) was used to determine whether the treatment produces a significant clinical improvement in pain (NRS) and function (RMDQ). The definition of MCID is ‘the smallest difference or change in a clinical outcome that is perceived as beneficial to patient’s medical management, assuming no excessive side effects and costs’.^42^ MCID is an evidence-based assessment tool for behavioural interventions across an entire procedure.^43,44^ A reduction of 2 or more in the NRS indicates positive clinical change.^45^ And several studies have recommended that the MCID for RMDQ-24 is the absolute cut-off of 5 points.^44,45^

10. The participants’ overall assessment of the treatment was measured on the basis of the global perceived effect (GPE). The GPE scale requires patients to rate how much they have improved or deteriorated since they received the treatment. The use of this scale is widely advocated in pain research because it is easy and quick to understand and score, and its results are important to patients.^46^ The most meaningful changes can be observed in patients answering the scale at a predefined time point. The GPE scale has excellent reproducibility (ICC = 0.90–0.99), and its correlation with disability (r = 0.40–0.74) is strong.^47^

11. Adverse events were collected from participants’ daily record forms. All pain (LBP or other pain) related to intervention or unrelated to intervention occurring during the research duration was considered.

12. The participants’ recommendation levels on the intervention that they received were classified as highly recommended, recommend, unclear, not recommended and strongly deprecated.

**Participant timeline**

The participants will be asked to complete the questionnaires personally at baseline, after 3 months of treatment, at 6-month follow-up, and at 12-month follow-up. During the follow-up period, the experimental team will contact the participants either via Wechat or phone regularly so that the data at the 6- and 12-month time points can be collected. Table 2 displays the items of the questionnaire and the measurement time points.

**Sample size calculation**

Sample size was calculated by G*power 3.1.9 based on the following conditions.

According to Costantino's trial, the subjects of the two groups received 12 weeks of aquatic exercise and back school program respectively. The effect size was calculated to be 0.35 by using the RMDQ score of the experimental group (mean = 5.37, SD = 1.82) and the control group (mean = 6.11, SD = 2.36) during the 3-month follow-up.^48^ The two groups were measured 4 times by using a mixed design of repeated measures analysis of variance. Considering that α = 0.05, power (1-β) = 0.95, Corr amongst rep measures = 0.5, the total sample size was 70. Considering the possibility of a 20% missing rate, the minimum sample size was 88. According to the calculation, 100 subjects will be expected to be recruited, and 50 subjects will be included in each group.

**Statistical analysis**

Data will be collected and analyzed via SPSS 20.0 and Microsoft 2016. Considering that someone may drop out midway, all outcomes should be conducted by intention-to-treat analysis and per-protocol analysis. For baseline variables, we will detect the categorical variables (e.g. gender, education levels and occupation) with chi-square test and analyze the continuous variables (e.g. age, height, weight, BMI, and pain intensity) with independent t-test or Mann–Whitney U test to compare the statistical significance between the TAE group and the PTMs group. The results of descriptive statistics will be presented as mean ± standard deviation.

The experimental results were compared through adjusted two-way repeated measures ANOVA (group × time). The adjustment factors included gender, age, BMI, physical activity, LBP duration, NRS of the most severe LBP, medication, and smoking history. A chi-square test was conducted to compare the proportion of participants in each group who met the MCID for pain and function at postintervention. Although the MCID for RMDQ and NRS remains controversial, values of 5.0 and 2.0 are considered reasonable and commonly used. The χ2 test was also applied to determine the difference between the 2 groups for the proportion of participants reporting GPE, adverse events and treatment recommendations. The effect sizes were presented to measure the strength of any outcome indicators, where 0.2 ≤ Cohen’s d < 0.5 means a ‘small’ effect size, 0.5 ≤ Cohen’s d < 0.8 indicates a ‘medium’ effect size and Cohen’s d ≥ 0.8 reflects a ‘large’ effect size.

**Discussion**

With various properties of water, TAE should be an acceptable and effective treatment for CLBP. In TAE usually performed in a group, creating high compliance and great effects on pain, people with CLBP interact with others with similar problems and receive peer support.^9,11,49^ TAE is a proactive and enjoyable treatment to convey the positive health concept naturally,^50^ which will cause long-term effects by avoiding unhealthy behaviors, such as absence from work and prolonged rest.^11,51^ Compared to PTMs, TAE can be carried out by patients themselves, which will cut down the medical expenditure to some extent.

To the best of our knowledge, this work is the first to compare the effect between TAE and PTMs on patients with CLBP. Thus, our study may also have the potential to optimize the strength of the water exercise, and our findings may provide broad functional and psychological benefits to practical applications.

**Strengths and limitations**

First, the research duration of most previous studies typically ranged from several weeks to 2 months.^22,23,52-54^ This study will include an intervention period of 3 months and a follow-up period (without intervention) of 3 and 9 months, making the entire term of 1 year. Second, our study will offer the same attention to all participants by designing the two programs with equal time, thereby reducing other biases compared with former studies.^55^ Third, we will add some psychological scales and lifestyle-related issues into our measurement. Thus, the CLBP will be accessed in a multi-tiered system.

Nevertheless, this method is not the perfect protocol. On the one hand, recall bias in questionnaire responses is inevitable, but we do not have enough data to show this influence. On the other hand, included people may have CLBP with different durations and degrees. Controlling the disease progression that affects individual physical fitness is difficult.

In conclusion, this trial aims to investigate the effect of TAE on people with CLBP and determine whether TAE elicits better results thanPTMs. Our findings will provide patients with an enjoyable and effective way to recovery, lessen the medical burden of CLBP, and change the public health and prevention strategies worldwide.^55,56^(61, 62)

**References**

1. Hartvigsen J, Hancock MJ, Kongsted A, et al. What low back pain is and why we need to pay attention. *Lancet (London, England).* 2018;391(10137):2356-2367.

2. Maher C, Underwood M, Buchbinder R. Non-specific low back pain. *Lancet (London, England).* 2017;389(10070):736-747.

3. Bernstein IA, Malik Q, Carville S, Ward S. Low back pain and sciatica: summary of NICE guidance. *BMJ (Clinical research ed).* 2017;356:i6748.

4. National Guideline C. National Institute for Health and Care Excellence: Clinical Guidelines. *Low Back Pain and Sciatica in Over 16s: Assessment and Management*. London: National Institute for Health and Care Excellence (UK) NICE, 2016(59).

5. Chou R, Deyo R, Friedly J, et al. Nonpharmacologic Therapies for Low Back Pain: A Systematic Review for an American College of Physicians Clinical Practice Guideline. *Ann Intern Med.* 2017;166(7):493-505.

6. Collaborators. GDaIIaP. Global, regional, and national incidence, prevalence, and years lived with disability for 328 diseases and injuries for 195 countries, 1990-2016: a systematic analysis for the Global Burden of Disease Study 2016. *Lancet (London, England).* 2017;390(10100):1211-1259.

7. Collaborators. GBoDS. Global, regional, and national incidence, prevalence, and years lived with disability for 301 acute and chronic diseases and injuries in 188 countries, 1990-2013: a systematic analysis for the Global Burden of Disease Study 2013. *Lancet (London, England).* 2015;386(9995):743-800.

8. Collaborators. GDaIIaP. Global, regional, and national incidence, prevalence, and years lived with disability for 310 diseases and injuries, 1990-2015: a systematic analysis for the Global Burden of Disease Study 2015. *Lancet (London, England).* 2016;388(10053):1545-1602.

9. Qaseem A, Wilt TJ, McLean RM, Forciea MA. Noninvasive Treatments for Acute, Subacute, and Chronic Low Back Pain: A Clinical Practice Guideline From the American College of Physicians. *Ann Intern Med.* 2017;166(7):514-530.

10. Clark S, Horton R. Low back pain: a major global challenge. *Lancet (London, England).* 2018;391(10137):2302.

11. Buchbinder R, van Tulder M, Oberg B, et al. Low back pain: a call for action. *Lancet (London, England).* 2018;391(10137):2384-2388.

12. Wenger HC, Cifu AS. Treatment of Low Back Pain. *Jama.* 2017;318(8):743-744.

13. Khadilkar A, Odebiyi DO, Brosseau L, Wells GA. Transcutaneous electrical nerve stimulation (TENS) versus placebo for chronic low-back pain. *The Cochrane database of systematic reviews.* 2008(4):Cd003008.

14. Lai CH, Leung TK, Peng CW, et al. Effects of far-infrared irradiation on myofascial neck pain: a randomized, double-blind, placebo-controlled pilot study. *J Altern Complement Med.* 2014;20(2):123-129.

15. Lahart IM, Metsios GS. Chronic Physiological Effects of Swim Training Interventions in Non-Elite Swimmers: A Systematic Review and Meta-Analysis. *Sports medicine (Auckland, NZ).* 2018;48(2):337-359.

16. Baena-Beato PA, Arroyo-Morales M, Delgado-Fernandez M, Gatto-Cardia MC, Artero EG. Effects of different frequencies (2-3 days/week) of aquatic therapy program in adults with chronic low back pain. A non-randomized comparison trial. *Pain medicine (Malden, Mass).* 2013;14(1):145-158.

17. Dundar U, Solak O, Yigit I, Evcik D, Kavuncu V. Clinical effectiveness of aquatic exercise to treat chronic low back pain: a randomized controlled trial. *Spine.* 2009;34(14):1436-1440.

18. Rivas Neira S, Pasqual Marques A, Pegito Perez I, Fernandez Cervantes R, Vivas Costa J. Effectiveness of Aquatic Therapy vs Land-based Therapy for Balance and Pain in Women with Fibromyalgia: a study protocol for a randomised controlled trial. *BMC musculoskeletal disorders.* 2017;18(1):22.

19. Bressel E, Dolny DG, Gibbons M. Trunk muscle activity during exercises performed on land and in water. *Medicine and science in sports and exercise.* 2011;43(10):1927-1932.

20. Carroll LM, Volpe D, Morris ME, Saunders J, Clifford AM. Aquatic Exercise Therapy for People With Parkinson Disease: A Randomized Controlled Trial. *Archives of physical medicine and rehabilitation.* 2017;98(4):631-638.

21. Shi Z, Zhou H, Lu L, et al. Aquatic Exercises in the Treatment of Low Back Pain: A Systematic Review of the Literature and Meta-Analysis of Eight Studies. *American journal of physical medicine & rehabilitation.* 2018;97(2):116-122.

22. Barker AL, Talevski J, Morello RT, Nolan GA, De Silva RD, Briggs AM. Jumping into the deep-end: results from a pilot impact evaluation of a community-based aquatic exercise program. *Clinical rheumatology.* 2016;35(6):1593-1601.

23. Nguyen C, Boutron I, Rein C, et al. Intensive spa and exercise therapy program for returning to work for low back pain patients: a randomized controlled trial. *Scientific reports.* 2017;7(1):17956.

24. Karagulle M, Karagulle MZ. Effectiveness of balneotherapy and spa therapy for the treatment of chronic low back pain: a review on latest evidence. *Clinical rheumatology.* 2015;34(2):207-214.

25. Cuesta-Vargas AI, Adams N, Salazar JA, Belles A, Hazanas S, Arroyo-Morales M. Deep water running and general practice in primary care for non-specific low back pain versus general practice alone: randomized controlled trial. *Clinical rheumatology.* 2012;31(7):1073-1078.

26. Cuesta-Vargas AI, White M, Gonzalez-Sanchez M, Kuisma R. The optimal frequency of aquatic physiotherapy for individuals with chronic musculoskeletal pain: a randomised controlled trial. *Disability and rehabilitation.* 2015;37(4):311-318.

27. Li F, Harmer P, Fitzgerald K, et al. Tai chi and postural stability in patients with Parkinson's disease. *The New England journal of medicine.* 2012;366(6):511-519.

28. Wallace LK, Slattery KM, Coutts AJ. The ecological validity and application of the session-RPE method for quantifying training loads in swimming. *Journal of strength and conditioning research.* 2009;23(1):33-38.

29. Elserty N, Kattabei O, Elhafez H. Effect of Fixed Versus Adjusted Transcutaneous Electrical Nerve Stimulation Amplitude on Chronic Mechanical Low Back Pain. *J Altern Complement Med.* 2016;22(7):557-562.

30. Khadilkar A, Odebiyi DO, Brosseau L, Wells GA. Transcutaneous electrical nerve stimulation (TENS) versus placebo for chronic low-back pain. *The Cochrane database of systematic reviews.* 2008(4):CD003008.

31. Diab AA, Moustafa IM. Lumbar lordosis rehabilitation for pain and lumbar segmental motion in chronic mechanical low back pain: a randomized trial. *J Manipulative Physiol Ther.* 2012;35(4):246-253.

32. Gale GD, Rothbart PJ, Li Y. Infrared therapy for chronic low back pain: a randomized, controlled trial. *Pain Res Manag.* 2006;11(3):193-196.

33. Morone N, Rollman B, Moore C, Li Q, Weiner D. A Mind-Body Program for Older Adults with Chronic Low Back Pain: Results of a Pilot Study. *Pain medicine (Malden, Mass).* 2009;10:1395-1407.

34. Mbada CE, Idowu OA, Ogunjimi OR, et al. Cross-cultural Adaptation, Reliability, and Validity of the Yoruba Version of the Roland-Morris Disability Questionnaire. *Spine.* 2017;42(7):497-503.

35. Yi H, Ji X, Wei X, et al. Reliability and validity of simplified Chinese version of Roland-Morris questionnaire in evaluating rural and urban patients with low back pain. *PLoS One.* 2012;7(1):e30807.

36. Zhang Y, Qu B, Lun SS, Guo Y, Liu J. The 36-item short form health survey: reliability and validity in Chinese medical students. *Int J Med Sci.* 2012;9(7):521-526.

37. Samakouri M, Bouhos G, Kadoglou M, Giantzelidou A, Tsolaki K, Livaditis M. Standardization of the Greek version of Zung's Self-rating Anxiety Scale (SAS). *Psychiatrike.* 2012;23(3):212-220.

38. Hita-Contreras F, Martinez-Lopez E, Latorre-Roman PA, Garrido F, Santos MA, Martinez-Amat A. Reliability and validity of the Spanish version of the Pittsburgh Sleep Quality Index (PSQI) in patients with fibromyalgia. *Rheumatol Int.* 2014;34(7):929-936.

39. Zhou XY, Xu XM, Wang F, et al. Validations and psychological properties of a simplified Chinese version of pain anxiety symptoms scale (SC-PASS). *Medicine.* 2017;96(10):e5626.

40. Aguiar AS, Bataglion C, Visscher CM, Bevilaqua Grossi D, Chaves TC. Cross-cultural adaptation, reliability and construct validity of the Tampa scale for kinesiophobia for temporomandibular disorders (TSK/TMD-Br) into Brazilian Portuguese. *Journal of oral rehabilitation.* 2017;44(7):500-510.

41. Wei X, Xu X, Zhao Y, Hu W, Bai Y, Li M. The Chinese version of the Tampa Scale for Kinesiophobia was cross-culturally adapted and validated in patients with low back pain. *J Clin Epidemiol.* 2015;68(10):1205-1212.

42. Fishbain DA, Gao J, Lewis JE, Zhang L. At Completion of a Multidisciplinary Treatment Program, Are Psychophysical Variables Associated with a VAS Improvement of 30% or More, a Minimal Clinically Important Difference, or an Absolute VAS Score Improvement of 1.5 cm or More? *Pain medicine (Malden, Mass).* 2016;17(4):781-789.

43. Grant S, Pedersen ER, Osilla KC, Kulesza M, D'Amico EJ. It is time to develop appropriate tools for assessing minimal clinically important differences, performance bias and quality of evidence in reviews of behavioral interventions. *Addiction (Abingdon, England).* 2016;111(9):1533-1535.

44. Lee MK, Yost KJ, McDonald JS, Dougherty RW, Vine RL, Kallmes DF. Item response theory analysis to evaluate reliability and minimal clinically important change of the Roland-Morris Disability Questionnaire in patients with severe disability due to back pain from vertebral compression fractures. *Spine J.* 2017;17(6):821-829.

45. Ostelo RW, Deyo RA, Stratford P, et al. Interpreting change scores for pain and functional status in low back pain: towards international consensus regarding minimal important change. *Spine.* 2008;33(1):90-94.

46. Evans R, Bronfort G, Maiers M, Schulz C, Hartvigsen J. "I know it's changed": a mixed-methods study of the meaning of Global Perceived Effect in chronic neck pain patients. *European spine journal : official publication of the European Spine Society, the European Spinal Deformity Society, and the European Section of the Cervical Spine Research Society.* 2014;23(4):888-897.

47. Kamper SJ, Ostelo RW, Knol DL, Maher CG, de Vet HC, Hancock MJ. Global Perceived Effect scales provided reliable assessments of health transition in people with musculoskeletal disorders, but ratings are strongly influenced by current status. *J Clin Epidemiol.* 2010;63(7):760-766.e761.

48. Costantino C, Romiti D. Effectiveness of Back School program versus hydrotherapy in elderly patients with chronic non-specific low back pain: a randomized clinical trial. *Acta Biomed.* 2014;85(3):52-61.

49. Lamb SE, Hansen Z, Lall R, et al. Group cognitive behavioural treatment for low-back pain in primary care: a randomised controlled trial and cost-effectiveness analysis. *Lancet (London, England).* 2010;375(9718):916-923.

50. Huber M, van Vliet M, Giezenberg M, et al. Towards a 'patient-centred' operationalisation of the new dynamic concept of health: a mixed methods study. *BMJ open.* 2016;6(1):e010091.

51. Auvinen J, Tammelin T, Taimela S, Zitting P, Karppinen J. Associations of physical activity and inactivity with low back pain in adolescents. *Scandinavian journal of medicine & science in sports.* 2008;18(2):188-194.

52. Backhausen MG, Tabor A, Albert H, Rosthoj S, Damm P, Hegaard HK. The effects of an unsupervised water exercise program on low back pain and sick leave among healthy pregnant women - A randomised controlled trial. *PLoS One.* 2017;12(9):e0182114.

53. Lambert BS, Greene NP, Carradine AT, et al. Aquatic treadmill training reduces blood pressure reactivity to physical stress. *Medicine and science in sports and exercise.* 2014;46(4):809-816.

54. Smith MA, Lyle MA. Chronic exercise decreases sensitivity to mu opioids in female rats: correlation with exercise output. *Pharmacology, biochemistry, and behavior.* 2006;85(1):12-22.

55. Koes B. Management of low back pain in primary care: a new approach. *Lancet (London, England).* 2011;378(9802):1530-1532.

56. Silvers WM, Rutledge ER, Dolny DG. Peak cardiorespiratory responses during aquatic and land treadmill exercise. *Medicine and science in sports and exercise.* 2007;39(6):969-975.

Supplement 1: Table 1 Protocol of Therapeutic Aquatic Exercise

| Activity/Exercise | Time (min) or REPs | Explanation | Modifications (easy/hard) | diagram | Equipment |
| --- | --- | --- | --- | --- | --- |
| Warm-up (10 min) | | | | | |
| Dynamic drafting | 4min, continuously  30 s × 8 parts | Stretching the muscles of the neck, shoulder, back, hip, knee, and ankle slowly and repeatedly to increase the range of motion of the entire joint. | Increase speed | 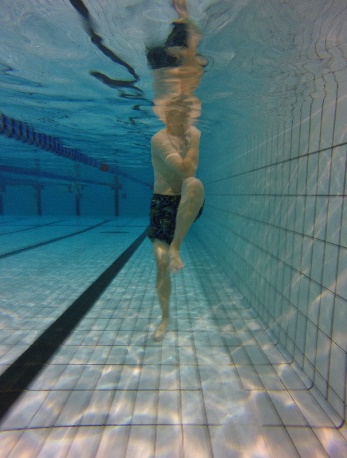 | / |
| Pool walking | 4 min, continuously | Walking in the water with tightening abdomen and straight back, and the heel touchdown before the front foot. | Increase speed | 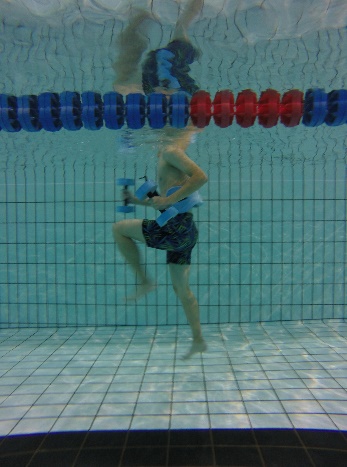 | / |
| High leg kicks | 1 min, continuously | Raising the legs alternately with fast rhythm. The body should be in the same position, and the upper body is upright. The heel of the supporting leg must be off the ground. | Increase speed | 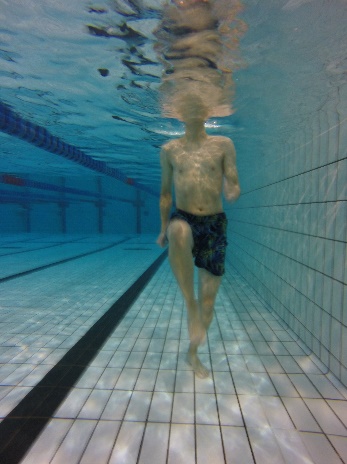 | / |
| Jumping jacks | 1 min, continuously | Standing in chest-level water with your feet together and your arms by your side. Jumping with two legs outward and arms lifted up to the head simultaneously, then bring the two legs together and put down the arms back to your side. | Increase speed | 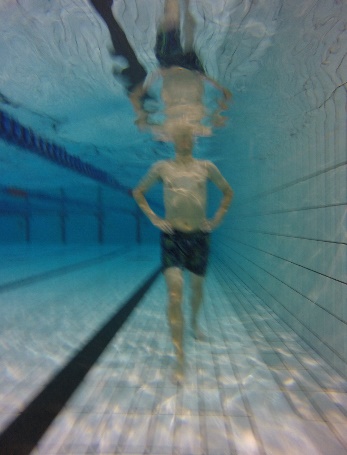 | / |
| Core exercise (40 min) | | | | | |
| Abdominal bracing | 5 min  15 s × 20reps  Maintain 10 s, rest 5 s | Standing still with your back in a relaxed neutral spine position, gently contracting your abdominal muscles to move the rib toward the hip and the navel to the spine. | Increase maintenance time | 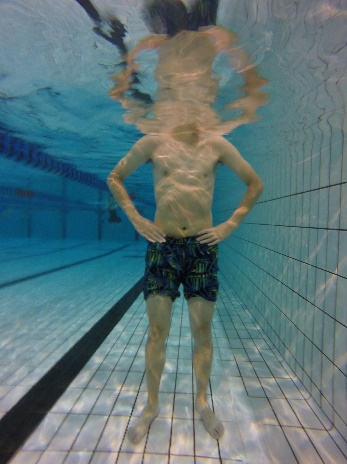 | / |
| Vertical downward press | 5 min  15 s × 20 reps  Maintain 10 s, rest 5 s | Standing upright with feet shoulder-width apart and toes forward, pressing the dumbbell vertical downward against the water with two hands. Then, the dumbbell was allowed to return to the surface slowly | Increase maintenance time, resistance | 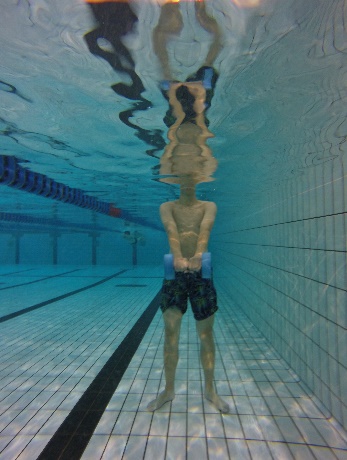 | Dumbbell、kickboard |
| Lateral downward press | 5min  15 s × 20 reps  Maintain 10 s, rest 5 s | Standing upright with the feet shoulder-width apart and toes forward, pressing the kickboard lateral downward against the water with the two hands. | Increase maintenance time, resistance | 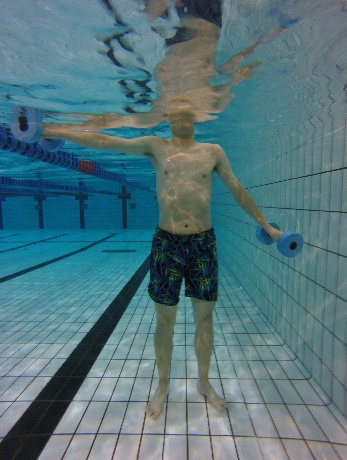 | Dumbbell, kickboard |
| Slant downward press | 10min  30 s × 20 reps×2direction  Maintain20 s, rest 10 s | Standing upright with feet shoulder-width apart and toes forward, holding the dumbbell with two hands in the direction of one shoulder, then pressing it slant downward against the water to the hip in another side. | Increase speed and repetitions | 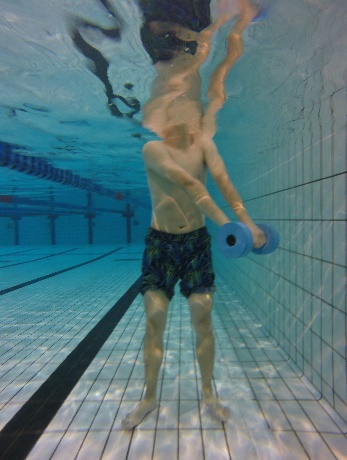 | Dumbbell, kickboard |
| Straight leg pressing | 5 min  30 s × 20 reps  Maintain20 s, rest 10 s | Standing with your back to the side of the pool in chest-high water, placing your arms on the edge of the pool for stability. Raising your two legs up together with straight knee joint, then return to standing position with two legs pressing against water. | Increase speed and repetitions, resistance | 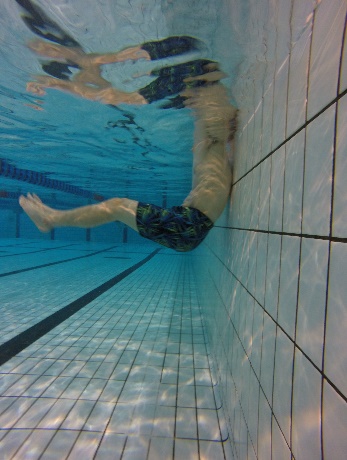 | Dumbbell, kickboard |
| Water treading | 5 mins  30 s × 20 reps  Maintain 20 s, rest 10 s | Standing in chin-high water with your feet held stationary on the top of a kickboard, paddle your arms to keep balance and kick your legs as your lower body rises off the ground and your knees to the chest. Then, extend your hips and knees fully to push the board reach the floor | Increase speed, maintain time | 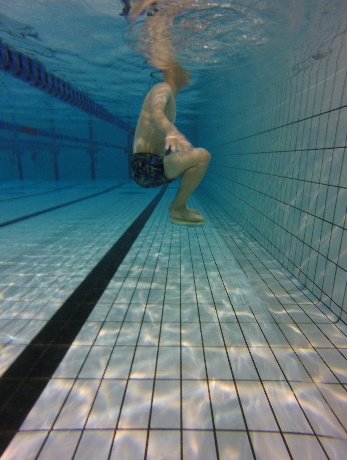 | kickboard |
| Deep water running | 5 min  Continuously run 50–100 m | A swim belt is needed to perform the exercise. Keep your body straight up in the water with your shoulders back and your head and eyes looking at the horizon. Pull your knees up as high as hip height, and slightly point your toes. Swing your arms with reciprocal boxing movements. The body will be propelled slowly forward | Increase speed and running mileage | 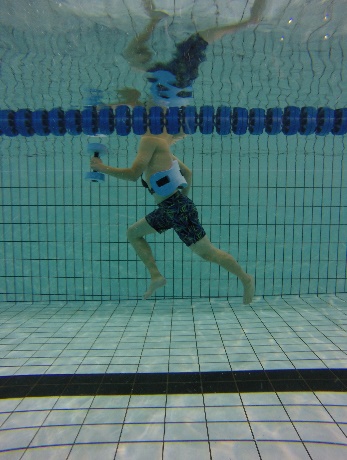 | Swim belt |
| Cool-down (10min) | | | | | |
| Static stretching | 5 min  20 s × 3 reps × 5 parts  Maintain 15 s, rest 5 s | Slowly lengthen the muscles of the shoulders, back, abdomen, and thighs, shins respectively, and keep them in a comfortable position for 15–20 s. | / | 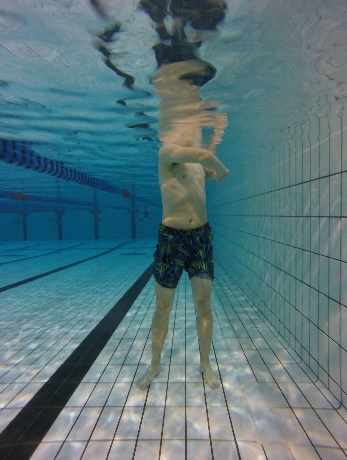 | / |
| Back float | 3 min  Continuously | Wearing a swim belt, lie as parallel to the ground as possible according to the instructions of the experimenter. Assistance given by the experimenter will be reduced gradually, and finally stop the assistance. | Decrease assistance | 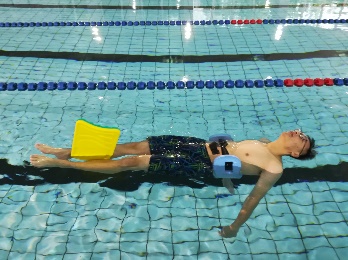 | Swim belt, kickboard |

Supplement 1: Table 2 demographics and baseline characteristics of all participants

| **Characteristic** | **therapeutic aquatic exercise** | **physical therapy modalities** |
| --- | --- | --- |
| Mean age (SD), y | ✖ | ✖ |
| Sex-female, N (%) | ✖ | ✖ |
| Mean height (SD), m | ✖ | ✖ |
| Mean weight (SD), kg | ✖ | ✖ |
| Mean BMI(SD), kg/m^2^ | ✖ | ✖ |
| **Education levels** |  |  |
| Illiteracy, N (%) | ✖ | ✖ |
| Primary school, N (%) | ✖ | ✖ |
| Junior middle school, N (%) | ✖ | ✖ |
| High school, N (%) | ✖ | ✖ |
| University, N (%) | ✖ | ✖ |
| Postgraduate, N (%) | ✖ | ✖ |
| **Employment status** |  |  |
| Employed part-time, No. (%) | ✖ | ✖ |
| Employed full-time, No. (%) | ✖ | ✖ |
| Unemployed, No. (%) | ✖ | ✖ |
| Not trying to look for employment, No. (%) | ✖ | ✖ |
| Unable to work due to poor health, No. (%) |  |  |
| Student, No. (%) | ✖ | ✖ |
| Retired, No. (%) | ✖ | ✖ |
| **Personal monthly income, yuan** | ✖ | ✖ |
| ≥10000, No. (%) | ✖ | ✖ |
| 5000-10000, No. (%) |  |  |
| 3000-5000, No. (%) | ✖ | ✖ |
| ＜3000, No. (%) | ✖ | ✖ |
| Refused, No. (%) | ✖ | ✖ |
| **Smoking history** |  |  |
| Smoking, No. (%) |  |  |
| Years of smoking, mean (SD), y | ✖ | ✖ |
| Smoking per day, mean (SD) | ✖ | ✖ |
| Low back pain duration, mean (SD), y | ✖ | ✖ |
| Duration of first onset, mean (SD),d | ✖ | ✖ |
| **Current back pain intensity** |  |  |
| Most serious pain in previous week, mean NRS score (SD) | ✖ | ✖ |
| Slightest pain in previous week, mean NRS score (SD) | ✖ | ✖ |
| Average pain in previous week, mean NRS score (SD) | ✖ | ✖ |
| Current pain intensity, mean NRS score (SD) | ✖ | ✖ |
| Work absence or reduced hours, mean (SD), h | ✖ | ✖ |
| Medical expenditure on back pain last year, mean (SD), yuan | ✖ | ✖ |
| **Medication use in previous three months** |  |  |
| No medication, No. (%) | ✖ | ✖ |
| Pain reliever, No. (%) | ✖ | ✖ |
| Adjuvant drugs, No. (%) | ✖ | ✖ |
| Drugs for other disease, No. (%) | ✖ | ✖ |
| **Belief that invention works** |  |  |
| Yes, No. (%) | ✖ | ✖ |
| No, No. (%) | ✖ | ✖ |
| Don't know, No. (%) | ✖ | ✖ |
| **Expectation that invention works** |  |  |
| Yes, No. (%) | ✖ | ✖ |
| No, No. (%) | ✖ | ✖ |
| Don't know, No. (%) | ✖ | ✖ |
| **Marital status** |  |  |
| Unmarried, No. (%) | ✖ | ✖ |
| Married, No. (%) | ✖ | ✖ |
| Divorced, No. (%) | ✖ | ✖ |
| Widowhood, No. (%) | ✖ | ✖ |
| **Occupation** |  |  |
| Sitting time , mean (SD), h | ✖ | ✖ |
| Standing time, mean (SD), h | ✖ | ✖ |
| Others, mean (SD), h | ✖ | ✖ |
| **Physical activity** |  |  |
| **High intensity physical activity per week** |  |  |
| ＜150min per day, No. (%) | ✖ | ✖ |
| 150-300min per day, No. (%) | ✖ | ✖ |
| ＞300min per day, No. (%) | ✖ | ✖ |
| **Moderate intensity physical activity per week** |  |  |
| ＜75min per day, No. (%) | ✖ | ✖ |
| 75-150min per day, No. (%) | ✖ | ✖ |
| ＞150min per day, No. (%) | ✖ | ✖ |
| **Cause of first onset** |  |  |
| Hyperactivity or improper exercise, No. (%) | ✖ | ✖ |
| Sedentary lifestyle, No. (%) | ✖ | ✖ |
| Pregnancy, No. (%) | ✖ | ✖ |
| Others, No. (%) | ✖ | ✖ |
| **Site of first onset** |  |  |
| Left, No. (%) | ✖ | ✖ |
| Right, No. (%) | ✖ | ✖ |
| Middle, No. (%) | ✖ | ✖ |
| Both two sides, No. (%) | ✖ | ✖ |
| Others, No. (%) | ✖ | ✖ |
| **Site of current LBP** |  |  |
| Left, No. (%) | ✖ | ✖ |
| Right, No. (%) | ✖ | ✖ |
| Middle, No. (%) | ✖ | ✖ |
| Both two sides, No. (%) | ✖ | ✖ |
| Others, No. (%) | ✖ | ✖ |
| Duration of the latest low back pain, mean (SD),d | ✖ | ✖ |
| Frequency of low back pain last month, mean (SD) | ✖ | ✖ |
| Duration of low back pain per day last week, mean (SD),h | ✖ | ✖ |
| **Influence of low back pain on work** |  |  |
| Free, No. (%) | ✖ | ✖ |
| mild, No. (%) | ✖ | ✖ |
| moderate, No. (%) | ✖ | ✖ |
| severe, No. (%) | ✖ | ✖ |
| **Influence of low back pain on life** |  |  |
| Free, No. (%) | ✖ | ✖ |
| mild, No. (%) | ✖ | ✖ |
| moderate, No. (%) | ✖ | ✖ |
| severe, No. (%) | ✖ | ✖ |
| **Pain mode in 24-hour** |  |  |
| gradually aggravate, No. (%) | ✖ | ✖ |
| gradually relieve, No. (%) | ✖ | ✖ |
| no change, No. (%) | ✖ | ✖ |
| Others, No. (%) | ✖ | ✖ |
| **Factors aggravating low back pain** |  |  |
| Sitting, No. (%) | ✖ | ✖ |
| Standing, No. (%) | ✖ | ✖ |
| Walking, No. (%) | ✖ | ✖ |
| Bending, No. (%) | ✖ | ✖ |
| Squat down, No. (%) | ✖ | ✖ |
| Go upstairs, No. (%) | ✖ | ✖ |
| Go downstairs, No. (%) | ✖ | ✖ |
| Postural change, No. (%) | ✖ | ✖ |
| Others, No. (%) | ✖ | ✖ |
| **Factors to relieve low back pain** |  |  |
| Recumbent rest, No. (%) | ✖ | ✖ |
| Sitting for rest, No. (%) | ✖ | ✖ |
| Small intensity activities, No. (%) | ✖ | ✖ |
| Others, No. (%) | ✖ | ✖ |
| **Nature of pain** |  |  |
| Soreness, No. (%) | ✖ | ✖ |
| Distended pain, No. (%) | ✖ | ✖ |
| Radiation pain, No. (%) | ✖ | ✖ |
| Burning pain, No. (%) | ✖ | ✖ |
| Needling pain, No. (%) | ✖ | ✖ |
| Other, No. (%) | ✖ | ✖ |

SD= Standard Deviation, BMI=Body Mass Index, VAS= Visual Analogue Scale, RMDQ= Roland Morris Disability Questionnaire

Supplement 1: Table 3 Outline of measurement time points

|  | Baseline | 3 months | 6 months | 12 months |
| --- | --- | --- | --- | --- |
| numeric rating scale (NRS) | ✖ | ✖ | ✖ | ✖ |
| Roland Morris Disability Questionnaire (RMDQ) | ✖ | ✖ | ✖ | ✖ |
| the Short Form (36) Health Survey | ✖ | ✖ | ✖ | ✖ |
| Self-Rating Anxiety Scale (SAS) | ✖ | ✖ | ✖ | ✖ |
| Zung Self-Rating Depression Scale (SDS) | ✖ | ✖ | ✖ | ✖ |
| Pittsburgh Sleep Quality Index (PSQI) | ✖ | ✖ | ✖ | ✖ |
| The Pain Anxiety Symptoms Scale (PASS) | ✖ | ✖ | ✖ | ✖ |
| Tampa Scale for Kinesiophobia (TSK) | ✖ | ✖ | ✖ | ✖ |
| Fear Avoidance Beliefs Questionnaire (FABQ) | ✖ | ✖ | ✖ | ✖ |
| The Minimal Clinically Important Difference (MCID) |  | ✖ | ✖ | ✖ |
| Global Perceived Effect (GPE) |  | ✖ | ✖ | ✖ |
| Adverse Events |  | ✖ | ✖ | ✖ |
| Recommendation |  | ✖ | ✖ | ✖ |
