# Supplementary material for: Effects of Therapeutic Aquatic Exercise Versus Physical Therapy Modalities on Pain and Disability in People With Chronic Low Back Pain: Potential Mediating Roles of Kinesiophobia, Anxiety, and Depression
Source: Pain Res Manag. 2026 Apr 12;2026:5537314. doi: 10.1155/prm/5537314 (PMC13071334; doi:10.1155/prm/5537314)
Supplement: Supplementary file 2 — Supporting Information 2 Supporting Figure 1. The mediating role of SDS on the effect that TAE had on RMDQ at 3‐month for subgroup with anxiety. ∗: p < 0.05, ∗∗: p < 0.01, ∗∗∗: p < 0.001. The path coefficients are regression coefficients. Abbreviations: TAE, therapeutic aquatic exercise; SDS, Zung Self‐Rating Depression Scale; RMDQ, Roland‐Morris Disability Questionnaire. [file PRM-2026-5537314-s004.docx]

Anxiety vs non-anxiety in TAE group

SDS-3mo

RMDQ-3mo

c'=-0.490

a=-13.896***

b=0.134**

c=-2.354*

a*b=-1.864*

**Supplementary Figure 1. The mediating role of SDS on the effect that TAE had on RMDQ at 3-month for subgroup with anxiety.** *: p < 0.05, **: p < 0.01, ***: p < 0.001. The path coefficients are regression coefficients. Abbreviations: TAE, therapeutic aquatic exercise; SDS, Zung self-rating depression scale; RMDQ, Roland-Morris Disability Questionnaire.
