# Supplementary material for: Effects of Therapeutic Aquatic Exercise Versus Physical Therapy Modalities on Pain and Disability in People With Chronic Low Back Pain: Potential Mediating Roles of Kinesiophobia, Anxiety, and Depression
Source: Pain Res Manag. 2026 Apr 12;2026:5537314. doi: 10.1155/prm/5537314 (PMC13071334; doi:10.1155/prm/5537314)
Supplement: Supplementary file 3 — Supporting Information 3 Supporting Figure 2. The mediating role of NRS average and NRS current on the effect that TAE had on TSK. Models showing the mediating role of NRS average on the relationship between TAE and TSK at 12‐month follow up (a); and the mediating role of NRS current on the relationship between TAE and TSK at 12‐month follow up (b); respectively. ∗: p < 0.05, ∗∗: p < 0.01, ∗∗∗: p < 0.001. The path coefficients are regression coefficients. Abbreviations: TAE, therapeutic aquatic exercise; NRS, Numeric Rating Scale; TSK, Tampa Scale for Kinesiophobia. [file PRM-2026-5537314-s003.docx]

TAE vs PTMs

NRS average-12mo

TSK-12mo

c'=-1.598

a=-1.451***

b=1.162**

TSK-12mo

NRS current-12mo

TAE vs PTMs

c'=-2.081

a=-1.360***

b=0.884*

c=-3.284*

c=-3.284*

a*b=-1.686*

a*b=-1.202*

a

b

**Supplementary Figure 2. The mediating role of NRS average and NRS current on the effect that TAE had on TSK.** Models showing the mediating role of NRS average on the relationship between TAE and TSK at 12-month follow up (a); and the mediating role of NRS current on the relationship between TAE and TSK at 12-month follow up (b); respectively. *: p < 0.05, **: p < 0.01, ***: p < 0.001. The path coefficients are regression coefficients. Abbreviations: TAE, therapeutic aquatic exercise; NRS, numeric rating scale; TSK, Tampa scale for kinesiophobia.
