# Supplementary material for: Effects of Therapeutic Aquatic Exercise Versus Physical Therapy Modalities on Pain and Disability in People With Chronic Low Back Pain: Potential Mediating Roles of Kinesiophobia, Anxiety, and Depression
Source: Pain Res Manag. 2026 Apr 12;2026:5537314. doi: 10.1155/prm/5537314 (PMC13071334; doi:10.1155/prm/5537314)
Supplement: Supplementary file 4 — Supporting Information 4 Supporting Figure 3. The mediating role of NRS average, NRS current and NRS most severe on the effect that TAE had on SDS at 12‐month follow up. Models showing the mediating role of NRS average on the relationship between TAE and SDS at 12‐month follow up (a); the mediating role of NRS current on the relationship between TAE and SDS at 12‐month follow up (b); and the mediating role of NRS slightest on the relationship between TAE and TSK at 12‐month follow up (c); respectively. ∗: p < 0.05, ∗∗: p < 0.01, ∗∗∗: p < 0.001. The path coefficients are regression coefficients. Abbreviations: TAE, therapeutic aquatic exercise; NRS, Numeric Rating Scale; SDS, Zung Self‐Rating Depression Scale. [file PRM-2026-5537314-s002.docx]

TAE vs PTMs

NRS most severe-12mo

SDS-12mo

c'=-3.130

a=-1.664***

b=1.242*

c=-5.196*

a*b=-2.066*

c

TAE vs PTMs

NRS average-12mo

SDS-12mo

c'=-2.835

a=-1.451***

b=1.626*

SDS-12mo

NRS current-12mo

TAE vs PTMs

c'=-2.334

a=-1.360***

b=2.105**

c=-5.196*

c=-5.196*

a*b=-2.361*

a*b=-2.861*

a

b

**Supplementary Figure 3. The mediating role of NRS average, NRS current and NRS most severe on the effect that TAE had on SDS at 12-month follow up.** Models showing the mediating role of NRS average on the relationship between TAE and SDS at 12-month follow up (a); the mediating role of NRS current on the relationship between TAE and SDS at 12-month follow up (b); and the mediating role of NRS slightest on the relationship between TAE and TSK at 12-month follow up (c); respectively. *: p < 0.05, **: p < 0.01, ***: p < 0.001. The path coefficients are regression coefficients. Abbreviations: TAE, therapeutic aquatic exercise; NRS, numeric rating scale; SDS, Zung self-rating depression scale.
