# Supplementary material for: Effects of Therapeutic Aquatic Exercise Versus Physical Therapy Modalities on Pain and Disability in People With Chronic Low Back Pain: Potential Mediating Roles of Kinesiophobia, Anxiety, and Depression
Source: Pain Res Manag. 2026 Apr 12;2026:5537314. doi: 10.1155/prm/5537314 (PMC13071334; doi:10.1155/prm/5537314)
Supplement: Supplementary file 5 — Supporting Information 5 Supporting Table 1. Subgroup analysis of the therapeutic effect of TAE on pain and dysfunction in patients with CLBP. Abbreviations: TAE, therapeutic aquatic exercise; CLBP, chronic low back pain; CI, confidence interval; NRS, Numeric Rating Scale; m, month; RMDQ, Roland‐Morris Disability Questionnaire. [file PRM-2026-5537314-s001.docx]

**Supplementary table 1****.** Subgroup analysis of the therapeutic effect of TAE on pain and dysfunction in patients with CLBP.

|  | **Grouping TAE based on anxiety** | | | |  | **Grouping TAE based on depression** | | | |
| --- | --- | --- | --- | --- | --- | --- | --- | --- | --- |
|  | **Anxiety group**  **SAS ≥ 50 (n=8)** | **Non-anxiety group**  **SAS < 50 (n=48)** | **p value** | **95%CI** |  | **Depression group**  **SDS ≥ 53 (n=13)** | **Non-depression group**  **SDS < 53 (n=43)** | **p value** | **95%CI** |
| NRS |  |  |  |  |  |  |  |  |  |
| Most severe |  |  |  |  |  |  |  |  |  |
| Baseline | 6.25(1.04) | 5.44(1.29) | 0.096 | -0.81(-1.77 to 0.15) |  | 6.23(1.48) | 5.35(1.15) | 0.028 | -0.88(-1.66 to -0.10) |
| 3 m | 3.25(2.19) | 2.60(1.43) | 0.279 | -0.65(-1.83 to 0.54) |  | 3.31(1.60) | 2.51(1.50) | 0.105 | -0.80(-1.76 to 0.17) |
| 6 m | 3.63(1.60) | 2.81(1.50) | 0.165 | -0.81(-1.97 to 0.34) |  | 3.15(1.21) | 2.86(1.61) | 0.548 | -0.29(-1.27 to 0.68) |
| 12 m | 4.13(1.46) | 3.00(1.65) | 0.076 | -1.13(-2.37 to 0.12) |  | 4.23(1.66) | 2.84(1.66) | 0.007 | -1.39(-2.39 to -0.40) |
| Average |  |  |  |  |  |  |  |  |  |
| Baseline | 4.50(0.93) | 3.88(1.16) | 0.154 | -0.63(-1.49 to 0.24) |  | 4.31(1.32) | 3.86(1.08) | 0.22 | -0.45(-1.17 to 0.27) |
| 3 m | 2.38(2.07) | 1.52(0.90) | 0.051 | -0.85(-1.71 to 0.004) |  | 2.23(1.54) | 1.47(0.96) | 0.11 | -0.77(-1.73 to 0.19) |
| 6 m | 2.63(0.92) | 1.98(1.10) | 0.123 | -0.65(-1.47 to 0.18) |  | 2.23(1.01) | 2.02(1.12) | 0.553 | -0.21(-0.91 to 0.49) |
| 12 m | 3.25(1.04) | 2.10(1.39) | 0.03 | -1.15(-2.18 to -0.11) |  | 3.08(1.26) | 2.02(1.35) | 0.016 | -1.05(-1.90 to -0.21) |
| Current |  |  |  |  |  |  |  |  |  |
| Baseline | 3.38(1.60) | 2.58(1.56) | 0.19 | -0.79(-1.99 to 0.40) |  | 3.00(1.78) | 2.60(1.51) | 0.432 | -0.40(-1.40 to 0.61) |
| 3 m | 1.50(1.41) | 0.85(0.90) | 0.09 | -0.65(-1.40 to 0.11) |  | 1.38(1.19) | 0.81(0.91) | 0.071 | -0.57(-1.19 to 0.05) |
| 6 m | 2.63(1.41) | 1.38(1.23) | 0.012 | -1.25(-2.21 to -0.29) |  | 1.85(1.72) | 1.47(1.18) | 0.367 | -0.38(-1.22 to 0.46) |
| 12 m | 2.00(1.07) | 1.42(1.35) | 0.251 | -0.58(-1.59 to 0.43) |  | 1.77(1.36) | 1.42(1.31) | 0.407 | -0.35(-1.19 to 0.49) |
| RMDQ |  |  |  |  |  |  |  |  |  |
| Baseline | 12.38(5.55) | 8.23(5.71) | 0.062 | -4.15(-8.50 to 0.21) |  | 11.46(5.71) | 8.02(5.68) | 0.061 | -3.44(-7.05 to 0.17) |
| 3 m | 5.25(3.96) | 2.90(2.60) | 0.032 | -2.35(-4.50 to -0.20) |  | 5.46(3.93) | 2.56(2.15) | 0.023 | -2.90(-4.59 to -1.22) |
| 6 m | 6.25(4.46) | 3.10(4.02) | 0.049 | -3.14(-6.27 to -0.02) |  | 5.62(4.86) | 2.93(3.82) | 0.042 | -2.69(-5.27 to -0.10) |
| 12 m | 6.63(5.85) | 3.00(4.00) | 0.031 | -3.63(-6.90 to -0.35) |  | 6.08(5.36) | 2.74(3.85) | 0.016 | -3.33(-6.02 to -0.65) |

Abbreviations: TAE, therapeutic aquatic exercise; CLBP, chronic low back pain; CI, confidence interval; NRS, numeric rating scale; m: month; RMDQ, Roland-Morris Disability Questionnaire.
