# Supplementary material for: Effects of Therapeutic Aquatic Exercise Versus Physical Therapy Modalities on Pain and Disability in People With Chronic Low Back Pain: Potential Mediating Roles of Kinesiophobia, Anxiety, and Depression
Source: Pain Res Manag. 2026 Apr 12;2026:5537314. doi: 10.1155/prm/5537314 (PMC13071334; doi:10.1155/prm/5537314)
Supplement: Supplementary file 6 — Supporting Information 6 Supporting Table 2. Direct and total effect coefficients and the results of bootstrapping (pain intensity as mediators). Abbreviations: X, independent variable (intervention variable); M = mediator; Y, dependent variable (outcome); CI, confidence interval; SE, standard error; TAE, therapeutic aquatic exercise; PTMs, physical therapy modalities; NRS, Numeric Rating Scale; m, month; TSK, Tampa Scale for Kinesiophobia; SDS, Zung Self‐Rating Depression Scale; SAS, Self‐Rating Anxiety Scale. ∗ p ≤ 0.05; ∗∗ p ≤ 0.01; ∗∗∗ p ≤ 0.001. [file PRM-2026-5537314-s006.docx]

**Supplementary Table 2.** Direct and total effect coefficients and the results of bootstrapping (pain intensity as mediators).

| Independent  variable (X) intervention variable | Mediator (M) | Dependent  variable (Y) | Coefficient a | Coefficient b | Coefficient c′ (direct effect) | Total effect c | Indirect effect (a*b) | | Bias-corrected 95% CI | | | Effect size |
| --- | --- | --- | --- | --- | --- | --- | --- | --- | --- | --- | --- | --- |
|  |  |  |  |  |  |  | Point estimate | SE | | Lower | Upper |  |
| TAE vs PTMs | NRS average | TSK (12 m) | -1.451*** | 1.162** | -1.598 | -3.284* | -1.686* | 0.662 | | -3.099 | -0.562 | 0.513 |
| TAE vs PTMs | NRS current | TSK (12 m) | -1.360*** | 0.884* | -2.081 | -3.284* | -1.202* | 0.552 | | -2.414 | -0.232 | 0.366 |
| TAE vs PTMs | NRS average | SDS (12 m) | -1.451*** | 1.626* | -2.835 | -5.196* | -2.361* | 1.239 | | -5.069 | -0.277 | 0.454 |
| TAE vs PTMs | NRS current | SDS (12 m) | -1.360*** | 2.105** | -2.334 | -5.196* | -2.861* | 1.283 | | -5.728 | -0.686 | 0.551 |
| TAE vs PTMs | NRS most severe | SDS (12 m) | -1.664*** | 1.242* | -3.130 | -5.196* | -2.066* | 1.218 | | -4.815 | -0.125 | 0.398 |

Abbreviations: X, independent variable (intervention variable); M = mediator; Y, dependent variable (outcome); CI, confidence interval; SE, standard error; TAE, therapeutic aquatic exercise; PTMs, physical therapy modalities; NRS, numeric rating scale; m: month; TSK, Tampa scale for kinesiophobia; SDS, Zung self-rating depression scale; SAS, self-rating anxiety scale. *p ≤ 0.05; **p ≤ 0.01; ***p ≤ 0.001.
